# Protein kinase A negatively regulates Ca^2+^ signalling in *Toxoplasma gondii*

**DOI:** 10.1371/journal.pbio.2005642

**Published:** 2018-09-12

**Authors:** Alessandro D. Uboldi, Mary-Louise Wilde, Emi A. McRae, Rebecca J. Stewart, Laura F. Dagley, Luning Yang, Nicholas J. Katris, Sanduni V. Hapuarachchi, Michael J. Coffey, Adele M. Lehane, Cyrille Y. Botte, Ross F. Waller, Andrew I. Webb, Malcolm J. McConville, Christopher J. Tonkin

**Affiliations:** 1 The Walter and Eliza Hall Institute of Medical Research, Parkville, Australia; 2 Department of Medical Biology, The University of Melbourne, Parkville, Australia; 3 School of Medicine, Tsinghua University, Beijing, China; 4 Department of Biochemistry, University of Cambridge, Cambridge, United Kingdom; 5 Institute of Advanced Biosciences, CNRS UMR5309, INSERM U1209, Université Grenoble Alpes, Grenoble, France; 6 Research School of Biology, The Australian National University, A.C.T., Australia; 7 Department of Biochemistry and Molecular Biology, Molecular Science and Biotechnology Institute, The University of Melbourne, Parkville, Australia; University of South Florida, United States of America

## Abstract

The phylum Apicomplexa comprises a group of obligate intracellular parasites that alternate between intracellular replicating stages and actively motile extracellular forms that move through tissue. Parasite cytosolic Ca^2+^ signalling activates motility, but how this is switched off after invasion is complete to allow for replication to begin is not understood. Here, we show that the cyclic adenosine monophosphate (cAMP)-dependent protein kinase A catalytic subunit 1 (PKAc1) of *Toxoplasma* is responsible for suppression of Ca^2+^ signalling upon host cell invasion. We demonstrate that PKAc1 is sequestered to the parasite periphery by dual acylation of PKA regulatory subunit 1 (PKAr1). Upon genetic depletion of PKAc1 we show that newly invaded parasites exit host cells shortly thereafter, in a perforin-like protein 1 (PLP-1)-dependent fashion. Furthermore, we demonstrate that loss of PKAc1 prevents rapid down-regulation of cytosolic [Ca^2+^] levels shortly after invasion. We also provide evidence that loss of PKAc1 sensitises parasites to cyclic GMP (cGMP)-induced Ca^2+^ signalling, thus demonstrating a functional link between cAMP and these other signalling modalities. Together, this work provides a new paradigm in understanding how *Toxoplasma* and related apicomplexan parasites regulate infectivity.

## Author summary

Central to pathogenesis and infectivity of *Toxoplasma* and related parasites is their ability to move through tissue, invade host cells, and establish a replicative niche. Ca^2+^-dependent signalling pathways are important for activating motility, host cell invasion, and egress, yet how this signalling is turned off after invasion is unclear. Here, we show that a cAMP-dependent protein kinase A (PKA) is essential for rapid suppression of Ca^2+^ signalling upon completion of host cell invasion. Parasites lacking this kinase rapidly invoke an egress program to re-exit host cells, thus preventing the establishment of a stable infection. This finding therefore highlights the first factor required for *Toxoplasma* (and any related apicomplexan parasite) to switch from invasive to the replicative forms.

## Introduction

The phylum Apicomplexa comprises a large group of obligate intracellular parasites that cause many important human and livestock diseases and includes *Plasmodium* spp. (malaria), *Cryptosporidium* spp. (severe diarrhoea), and *Toxoplasma gondii* (toxoplasmosis). *Toxoplasma* is transmitted to humans by eating undercooked meat harbouring cyst forms or by consuming soil, vegetables, or water contaminated with oocysts shed from an infected cat. In healthy individuals, acute toxoplasmosis manifests with mild flu-like symptoms and is self-resolving; however, infection can cause life-threatening illness in the immunocompromised. Furthermore, infection during pregnancy can cause abortion early in gestation or severe neurological developmental abnormalities in the foetus. Vertical transmission is also considered the cause of the relatively high rates of progressive blindness in some countries [1,2].

*Toxoplasma*, like all apicomplexan parasites, critically requires the ability to move through tissue and invade host cells for survival and proliferation. Parasite movement is powered by a distinctive form of cellular locomotion referred to as ‘gliding motility’, which is powered by the glideosome, an actomyosin-dependent motor located underneath the plasma membrane [3,4]. Gliding motility is activated when transmembrane adhesins are released from the microneme organelles at the apical tip of the parasite and deposited onto the parasite surface. The current model then posits that the glideosome drives parasite motility when dynamic actin filaments attach to the cytoplasmic tails of transmembrane adhesins via the glideosome associated connector (GAC) [5]. This then allows Myosin A (MyoA), which is anchored in the parasite pellicle, to engage and drag actin-adhesin complexes rearward through the plane of the membrane, to the basal end, thus resulting in forward directional movement of the parasite through tissue and into host cells [3].

Parasite egress and motility are under tight control of intracellular signal transduction pathways so as to only activate at an appropriate time and switch off upon host cell invasion. Both microneme and glideosome activity are controlled by the cytosolic concentration of Ca^2+^ ([Ca^2+^]_cyt_), cyclic GMP (cGMP), and inositol signalling pathways [6–8]. The current model suggests that cGMP signalling is activated by extracellular signals and results in stimulation of inositol phosphate metabolism and subsequent release of Ca^2+^ from intracellular stores, as well as influx of Ca^2+^ from the external environment [9,10]. A rise in [Ca^2+^]_cyt_ is temporally linked to the activation of egress from host cells and subsequent bursts of extracellular motility [11–13]. A rise in [Ca^2+^]_cyt_ then triggers Ca^2+^-dependent protein kinases (CDPKs) and a calcineurin phosphatase, which then likely phosphorylate/dephosphorylate substrates, triggering downstream events, including activation of the glideosome and the release of adhesins from the micronemes [9,14–21].

To transition from immotile replicating tachyzoites to actively motile and invasive forms, parasites must sense the extracellular environment to regulate motility. High extracellular [K^+^], as encountered in the host cell, inactivates motility, whilst a drop in this cation causes a rise in [Ca^2+^]_cyt_ and activation of locomotion [22]. More recently, it has been shown that extracellular pH ([H^+^]) also regulates motility. Here, low extracellular pH potently activates motility, whilst also being able to overcome the suppressive effect of high extracellular [K^+^] [23]. During intracellular growth, a lowering of the pH of the vacuolar space can activate Ca^2+^-dependent egress whilst also promoting an acidic environment for the activation of perforin-like protein 1 (PLP-1) to elicit membrane damage and tachyzoite egress [23].

Despite advances in our understanding of how *Toxoplasma* and other apicomplexan parasites activate motility, it remains unclear how these important pathogens sense environmental cues and how Ca^2+^ signalling is switched off. In other eukaryotic systems, G-protein coupled receptors (GPCRs) are essential for environmental sensing and act by receiving extracellular cues and transducing these signals across the plasma membrane. GPCRs physically associate with signalling proteins on the cytoplasmic side of the plasma membrane, causing changes in their activity upon extracellular cues. GPCRs commonly activate cyclic adenosine monophosphate (cAMP) signalling pathways. This occurs by direct coupling of GPCRs to adenyl cyclases (ACs), which are activated to produce cAMP upon encountering environmental stimuli. cAMP then activates a tetrameric protein kinase A (PKA) complex by binding to the regulatory subunit (PKAr), promoting its dissociation from the catalytic subunit (PKAc) and thus promoting downstream phosphorylation. Whilst genome sequencing has shown a complete lack of GPCRs in Apicomplexa, previous work has suggested that PKA, and thus cAMP signalling, is important for parasite invasion in several stages of the *Plasmodium* life cycle, in part through the phosphorylation of the cytoplasmic tail of the parasite adhesin apical membrane antigen 1 (AMA1) [24–26] and may be linked to Ca^2+^ signalling [15]. Interestingly, one of three PKA paralogues in *Toxoplasma* negatively regulates bradyzoite differentiation [27].

Here, we show that protein kinase A catalytic subunit 1 (PKAc1) acts as a negative regulator of tachyzoite egress immediately following invasion and is required for the transition of invasive tachyzoites into intracellular replicative forms. Whilst PKAc1-depleted tachyzoites appear to invade normally and form a parasitophorous vacuole (PV), they rapidly egress from host cells shortly thereafter. We show that in the absence of PKAc1, newly invaded tachyzoites cannot rapidly quench [Ca^2+^]_cyt_, leading to a persistent raised level. Furthermore, we provide evidence that PKAc1 dampens cGMP signalling to supress [Ca^2+^]_cyt_. Together, our work pinpoints a critical role of PKAc1, and thus cAMP signalling, in negatively regulating [Ca^2+^]_cyt_ upon parasite invasion. Our work therefore provides evidence of how *Toxoplasma*, and potentially other apicomplexan parasites, switch off motility upon successful invasion of a host cell.

## Results

### A PKA complex in *Toxoplasma* localises to the parasite periphery

**Fig 1:**
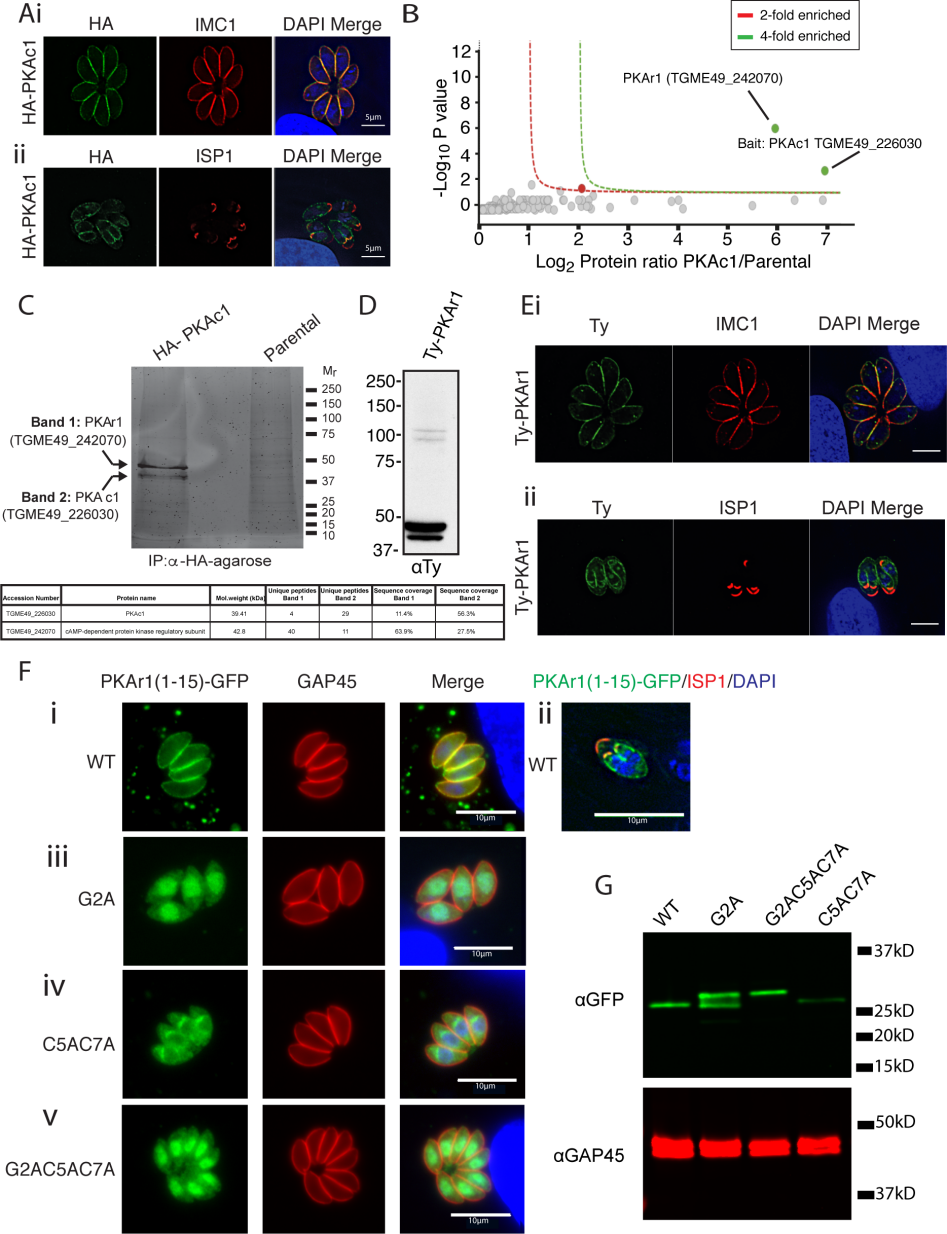
*Toxoplasma* PKAc1 localises to the tachyzoite periphery likely by dual acylation of its regulatory subunit PKAr1. **(**A) Localisation of HA-tagged PKAc1 by IFA, co-staining with the peripheral marker IMC1 (i) and the apical IMC marker ISP1 [30] (ii). (B) Identification of PKAc1-interacting proteins by anti-HA immunoprecipitation, whole eluate tryptic digestion, mass spectrometry, and label-free quantification. Red and green lines signify 2-fold and 4-fold enrichment, as compared to the parental line (see S1 Table for full list of identified proteins). (C) Identification of PKAc1-interacting proteins by anti-HA immunoprecipitation, SYPRO Ruby staining of eluates followed by in-gel digest and mass spectrometry. Table denotes proteins and coverage identified in each band. (D) Western blot of Ty epitope-tagged PKAr1 (TGME49_242070). (E) Localisation of Ty epitope tagged PKAr1 by IFA, co-stained with either (i) IMC1 antibodies or (ii) the antibodies to the apical IMC protein ISP1, outlining localisation to the developing daughter cells. (F) Mutational analysis of putative myristoylation and palmitoylation sites at the N-terminus of PKAr1. (i) The first 15 amino acids of PKAr1 (PKAr1(1–15)_WT_) were fused to GFP and colocalised with GAP45 and (ii) ISP1, showing localisation to the periphery and to the developing daughter cells, respectively. (iii) Putative myristoylation site—glycine at position 2—was mutated to alanine (PKAr1(1–15)_G2A_), (iv) putative palmitoylated cysteine residues at positions 5 and 7 were mutated to alanine (PKAr1(1–15)_C5AC7A_). (v) Both putative myristoylation and palmitoylation sites were mutated (PKAr1(1–15)_G2AC5AC7A_). In each case, localisation of GFP fusions were compared to the peripheral marker GAP45 and counterstained with DAPI. (G) Western blot on PKAr1(1–15)_WT_-GFP and point mutants. C5AC7A, cysteine-to-alanine-mutations at amino acid 5 and 7; GAP45, glideosome-associated protein 45; GFP, green fluorescent protein; G2A, glycine to alanine mutation at amino acid 2; HA, haemagglutinin; IFA, immunofluorescence assay; IMC, inner membrane complex; IP, immunoprecipitation; ISP1, IMC subcompartment protein 1; PKAc1, protein kinase A catalytic subunit 1; PKAr1, protein kinase A regulatory subunit 1; WT, wild type.

We wished to understand if cAMP/PKA signalling participates in relaying environmental cues to modulate motility. *Toxoplasma* contains three annotated PKA catalytic subunit paralogues (toxodb.org) [28]. Previous work has shown that PKAc3 (TGME49_286470) negatively regulates the differentiation of bradyzoite forms during tachyzoite growth [27], whilst PKAc2 (TGME49_228420) appears not expressed in tachyzoite stages (toxodb.org). PKAc1 (TGME49_226030) is the most widely conserved *Toxoplasma* paralogue across the Apicomplexa and thus was chosen for study. To understand the function of PKAc1, we genetically introduced an epitope tag at the 5′ end of the protein, whilst simultaneously putting the gene under conditional genetic control using the tetracycline-off (tet-off) system (S1 Fig) (see below for description). To assess the localisation of PKAc1, we performed an immunofluorescence assay (IFA) using anti-haemagglutinin (HA) epitope antibodies and observed a peripheral staining pattern, as marked by inner membrane complex 1 (IMC1) protein antibodies [29] (Fig 1Ai). In some tachyzoites, we noticed internal staining, which colocalised with the apical IMC sub-compartment protein 1 (ISP1) marker [30], suggesting association with the forming daughter cells during replication (Fig 1Aii).

Across eukaryotes, PKA typically localises to defined cellular compartments via association with A-kinase-associated proteins (AKAPs) and/or by binding to GPCRs. To determine if this was true in *Toxoplasma*, we performed immunoprecipitation of HA-tagged PKAc1 and compared eluates to parental controls either by in-solution trypsin digestion (Fig 1B) or in-gel digests from SYRPO Ruby-stained gels, followed by mass spectrometry to detect associated proteins (Fig 1C). In both cases we only confidently detected one additional protein, which we determined to be TGME49_242070, a protein with several predicted cAMP-binding domains. The *Toxoplasma* genome (toxoDB.org) predicts the presence of two regulatory PKA subunits (the other being TGME49_311300), and thus this work experimentally verifies that TGME49_242070 is most likely the regulatory PKA subunit for PKAc1 –thus we named this orthologue PKAr1.

PKAr subunits in other eukaryotes contain an N-terminal docking domain that is responsible for associating the PKA complex with AKAP. *Toxoplasma* PKAr1 appears to lack this domain and instead contains a glycine at position 2 and two cysteines at positions 5 and 7, typical of sites of acylation. Acylation is an important mechanism for imparting membrane affinity on a range of proteins involved in signalling and motility in *Toxoplasma* [31–34]. To investigate the localisation of PKAr1 without disturbing this potential motif, we introduced a Ty epitope tag 15 amino acids downstream from the starting methionine. Western blot of Ty-PKAr1 revealed that there were two major species of this protein close to the expected size, suggesting that dual acylation could be imparting changes to the migration of the protein (Fig 1D). There are also two weak bands at about 100 kD, but the identity or significance of these is not known. Colocalisation of Ty-PKAr1 by IFA with IMC1 also demonstrated a largely peripheral localisation and further, we also observed localisation to the nascent IMC (as marked by ISP1) during internal daughter cell budding (Fig 1Ei and 1Eii), similar to that observed with PKAc1.

PKAr1 was recently described to be part of the *Toxoplasma* ‘palmitome’, thus further supporting the evidence that this protein is acylated [35]. To investigate whether the N-terminal region of PKAr1 was responsible for sequestration to the periphery by myristoylation of glycine 2 (G2) and palmitoylation of cysteine 5 (C5) and/or 7 (C7), we fused the first 15 amino acids of PKAr1 to green fluorescent protein (GFP) and used IFA to monitor localisation and western blot to assess any changes in migration pattern. We found that, indeed, PKAr1(1–15) was sufficient to target GFP to the parasite periphery (Fig 1Fi) and to internal budding daughter cells (Fig 1Fii). Furthermore, mutation of the putative myristoylation site to an alanine (G2A) resulted in a severe abrogation of peripheral localisation and instead caused an accumulation of the GFP fusion protein in the cytoplasm and nucleus (Fig 1Fiii). Mutation of the two putative palmitoylation acceptor cysteines to alanines (C5A, C7A) also resulted in abrogation of peripheral localisation (Fig 1Fiv), as did mutation of all three residues simultaneously (G2A, C5A, C7A) (Fig 1Fv). Protein acylation leads to changes in hydrophobicity and propensity to bind detergents, leading to change in apparent molecular weight on SDS-PAGE. To further implicate myristoylation and palmitoylation in PKAr1 membrane affinity, we monitored changes in protein migration of PKAr1(1–15)_WT_-GFP and point mutants by western blot (Fig 1G). Here, we observed a single molecular weight species in PKAr1(1–15)_WT_-GFP, which, upon mutation of the glycine at position 2 to alanine (PKAr1(1–15)_G2A_-GFP), resulted in an additional higher molecular weight species, consistent with myristoylation causing faster migration (Fig 1G). Mutation of both putative myristoylation and palmitoylation sites (PKAr(1–15)_G2A C5A C7A_-GFP resulted in complete loss of faster migrating species (Fig 1G). Mutation of the putative palmitoylation sites (PKAr(1–15)_C5A C7A_-GFP) does not appear to affect SDS-PAGE migration as compared to the wild-type sequence, suggesting that myristoylation is able to occur in the absence of palmitoylation sites (Fig 1G). Together, these data are consistent with PKAc1 localising to the parasite periphery, likely the IMC membranes, via its association with PKAr1, which relies on dual acylation to derive membrane binding.

### PKAc1 is essential for the tachyzoite lytic cycle

**Fig 2:**
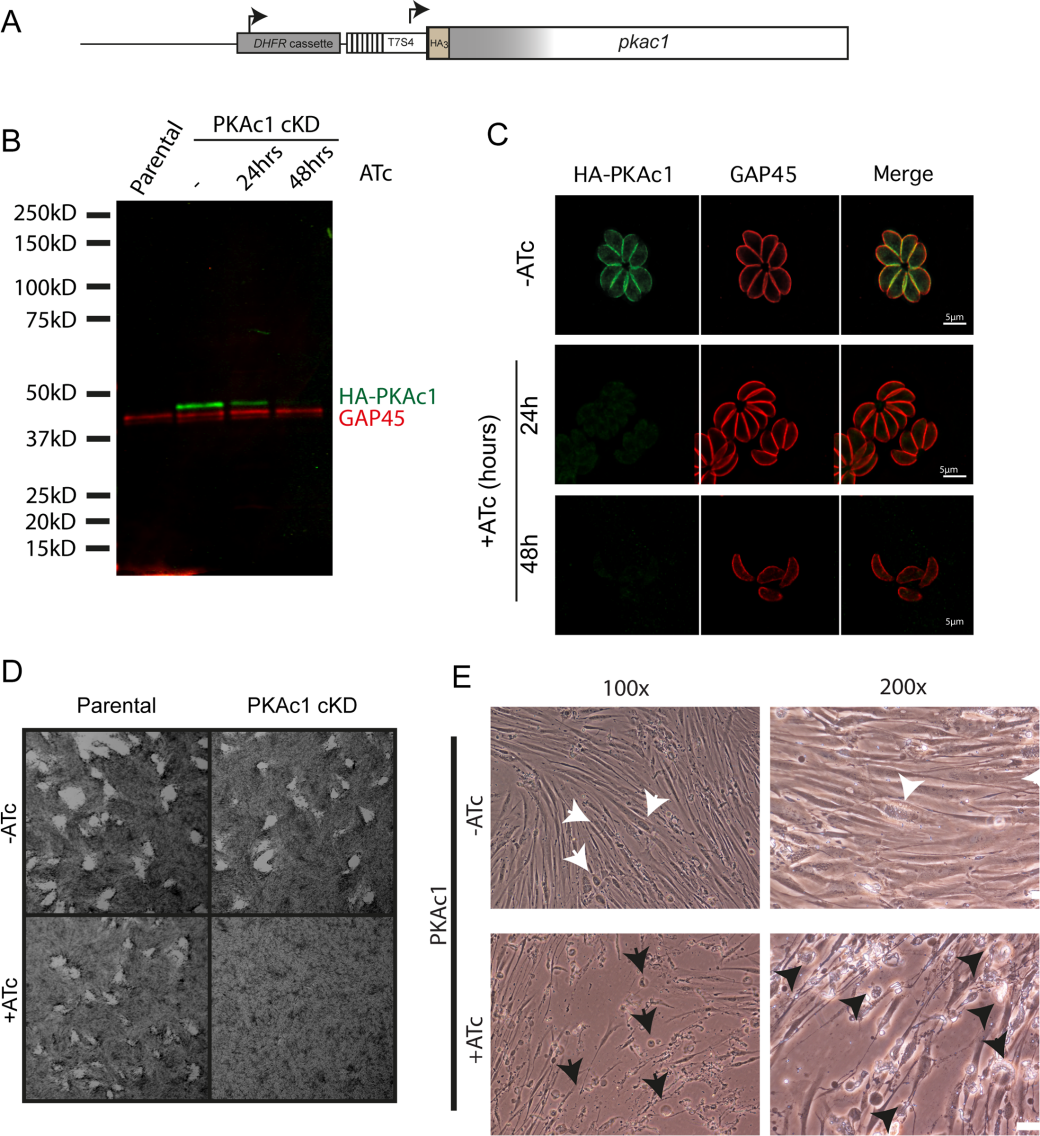
Generation and characterisation of a conditional knockdown of *Toxoplasma* PKAc1. (A) Schematic representation of tet-off promoter replacement-based conditional knockdown of PKAc1 (PKAc1 cKD). Details of genetic strategy and validation of PKAc1 cKD are available in S1 Fig. (B) Western blot of down-regulation of PKAc1 levels upon ATc treatment at 24 hours and 48 hours and comparison to parental line. HA antibodies were used to detect PKAc1 and GAP45 antibodies were used as a loading control. (C) IFA of PKAc1 upon ATc treatment for 24 hours and 48 hours. (D) Plaque assay of parental and PKAc1 cKD and parental lines ± ATc upon infection of confluent HFF monolayers. (E) Images of in vitro cultures of HFFs infected with PKAc1 cKD tachyzoites after for 24 hours growth ± ATc. White arrows point to intact intracellular parasite vacuoles and black arrows show examples of unhealthy and detached HFFs. Scale bars = 50 μm. More images are available in S2 Fig. ATc, anhydrotetracycline; cKD, conditional knockdown; DHFR, dihydrofolate reductase; GAP45, glideosome-associated protein 45; HA, haemagglutinin; HFF, human foreskin fibroblast; IFA, immunofluorescence assay; PKAc1, protein kinase A catalytic subunit 1.

To investigate the function of PKAc1 in *Toxoplasma* tachyzoites, we generated a PKAc1 conditional knockdown (cKD) line by replacing the endogenous promoter with the tet-off tetO7sag4 (T7S4) promoter (S1 Fig) (Fig 2A) [36,37]. To monitor the regulation of PKAc1 in the PKAc1 cKD parasites, we tracked protein production by the N-terminally appended HA epitope tag using HA antibodies. Treatment of parasite cultures with anhydrotetracycline (ATc) for 48 hours led to a marked decrease in PKAc1 levels (Fig 2B), and IFA showed loss of detectable levels of this protein (Fig 2C). To monitor growth of PKAc1 cKD parasites across the complete lytic cycle, we then performed plaque assays on confluent monolayers of human foreskin fibroblasts (HFFs). ATc treatment of PKAc1 cKD, but not parental, parasites resulted in a drastic reduction in plaque size, consistent with this kinase having an important role in one or more steps of the *Toxoplasma* lytic cycle, and furthermore is congruent with its deleterious clustered regularly interspaced short palindromic repeats (CRISPR) screen growth score [38] (Fig 2D). During our preliminary analysis, we noticed that in vitro cultures of PKAc1-depleted tachyzoites growing in host cells had an unusual presentation. After 24 hours of ATc treatment, PKAc1 cKD cultures contained very few intracellular parasites (compared to untreated cultures). Concurrently, host cells appeared to have been lysed, some detaching from the surface plastic (Fig 2E and S2 Fig).

### PKAc1-deficient tachyzoites re-exit host cells after invasion

**Fig 3:**
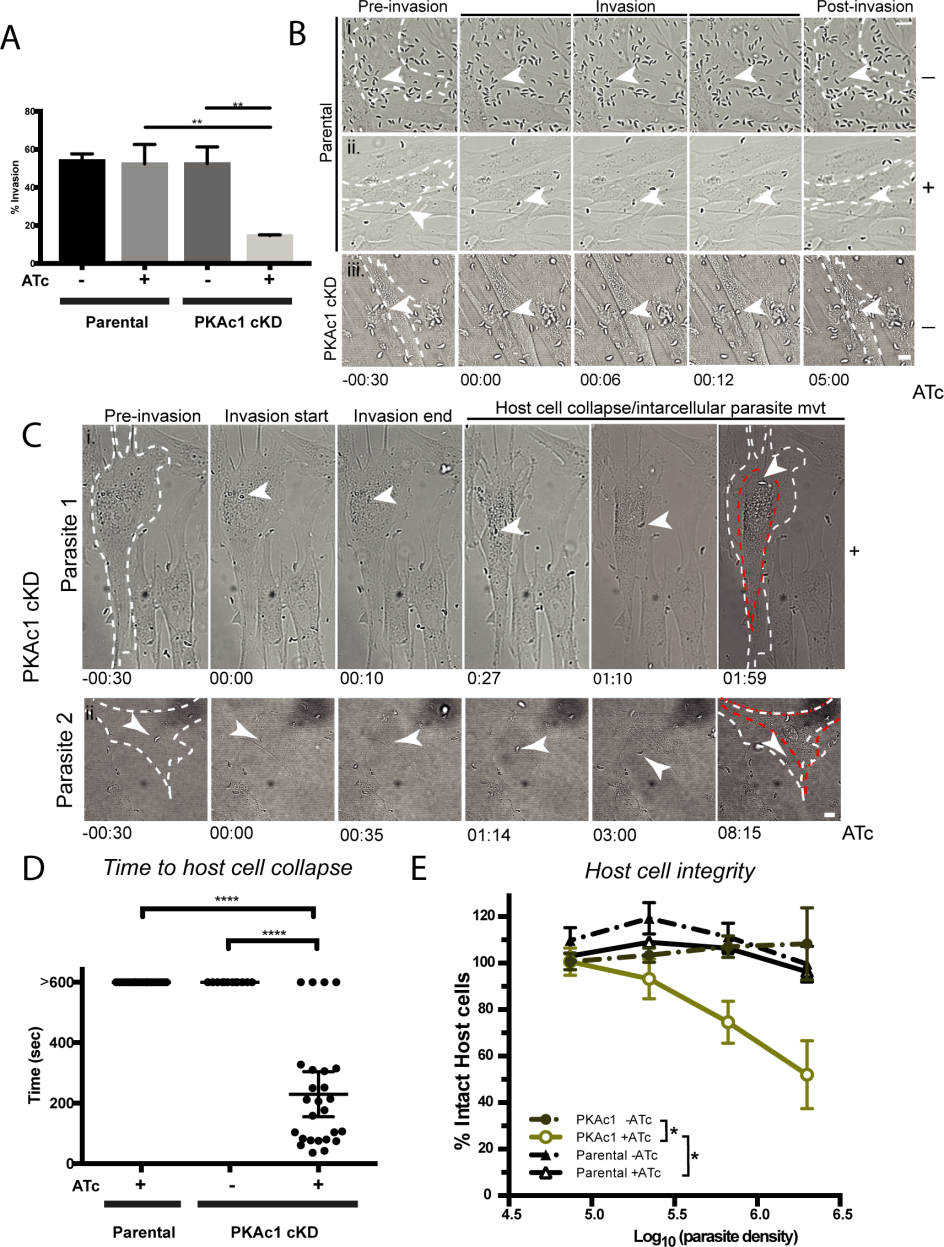
PKAc1-deficient *Toxoplasma* tachyzoites damage host cells upon invasion. (A) Static invasion assay of parental and PKAc1cKD tachyzoites. Data are the mean ± SEM of three biological replicates. For each replicate, parasites in 16 panels, each with an area of 211.3 × 198.1 μm, were counted at 63× magnification. *P* values are calculated using an unpaired two-tailed *t* test, where ** ≤ 0.005. (Bi and ii) Live cell imaging of parental strain ± ATc and (iii) PKAc1 cKD without ATc treatment before, during, and after invasion of host HFFs. White arrowheads point to invading tachyzoites and dashed white lines signify the border of the target HFF before and after invasion. Time in minutes and seconds is displayed across the bottom of panels. Stills correspond to S1, S2, and S3 Movies, respectively. Scale bar = 10 μm. (Ci and ii) Live cell imaging of two representative PKAc1-depleted tachyzoites (+ATc) before, during, and after invasion of HFFs. As above, white dashed lines outline host cell border pre-invasion, whilst red border denotes HFF outline post-invasion. White arrowheads signify tracked tachyzoites. Time in minutes and seconds is displayed across the bottom of panels. Stills correspond to S4 and S5 Movies. Scale bar = 10 μm. (D) Quantification of time to host cell collapse in Parental and PKAc1-depleted lines. Greater than 600 signifies that host cell collapse was not observed over the 10-minute (600-second) filming period. Data represent mean ± 95% confidence intervals; Parental + ATc, *n* = 20; PKAc1-ATc, *n* = 12; PKAc1 + ATc, *n* = 26 across at least three experiments. *P* values are calculated using an unpaired two-tailed *t* test, where **** ≤ 0.0001. (E) Integrity of host cells invaded for 1 hour by parental and PKAc1cKD lines, ±ATc as measured as a function of tachyzoite number versus crystal violet absorbance. Data represent mean ± SEM of four technical replicates. *P* values are calculated using a two-way ANOVA, where * ≤ 0.05 only at highest tachyzoite concentration. Individual numerical values underlying (A), (D), and (E) may be found in S1 Data. ATc, anhydrotetracycline; cKD, conditional knockdown; HFF, human foreskin fibroblast; PKAc1, protein kinase A catalytic subunit 1; PKAc1cKD, protein kinase A catalytic subunit 1 conditional knockdown.

We hypothesised that the unusual presentation of host cells in cultures infected with PKAc1-deficient tachyzoites was due to aberrant invasion. We therefore performed two-colour invasion assay to enumerate invasion efficiency on a population level. After a 10-minute invasion period we observed a stark loss of intracellular parasites (Fig 3A). To get a better understanding of the role of PKAc1 during invasion we undertook live cell imaging. In ATc-treated or untreated parental lines and untreated PKAc1 cKD tachyzoites we saw typical invasion, in which parasites attached to host cell monolayers, activated motility, and penetrated the host cell through a tight constriction, typical of the formation of the moving junction (Fig 3Bi, Bii and 3Biii; S1, S2 and S3 Movies). Following invasion, tachyzoites remained stationary and little movement of the host cell was observed (invading tachyzoites marked with white arrowhead and dashed white lines mark outline of host cell) (Fig 3Bi, 3Bii and 3Biii; S1, S2 and S3 Movies). Upon depletion of PKAc1, shortly after the apparent completion of invasion, we saw that the host cell began to detach from the glass slide surface, often with the concomitant reactivation of parasite motility (Fig 3Ci and 3Cii, S4 and S5 Movies). We observed that tachyzoites could directly exit host cells (Fig 3Cii, S4 Movie) and in some cases move within the confines of the damaged host cell (Fig 3Ci, S5 Movie). We then quantified the timing of host cell collapse in relation to invasion over a larger population of cells. Over a period of 10 minutes of filming we saw no parental (+ATc) or PKAc1 cKD (−ATc) egress (Fig 3D). In comparison, some PKAc1-deficient tachyzoites exited as quickly as about 30 seconds post-invasion, whereas others did not exit within the 10-minute filming period. Over the population, host cell collapse occurred at an average of about 200 seconds post-invasion (Fig 3D). We then quantified host cell damage on a population level, as a function of parasite concentration, by monitoring host cell integrity using crystal violet staining. To do this, ATc treated and untreated parental and PKAc1 cKD tachyzoites were serially diluted and allowed to invade host cells for 3 hours, after which time host cell integrity was analysed by crystal violet staining (Fig 3E). The number of intact host cells decreased when incubated with increasing concentration of PKAc1-depleted tachyzoites. In contrast, PKAc1 cKD parasites expressing PKAc1 and parental parasites treated with ATc caused no such damage (Fig 3E). Together, these results suggest that PKAc1 is required for productive invasion and/or the suppression of motility once cell entry is complete.

### PKAc1 does not play a detectable role during invasion

The exit of host cells by PKAc1-depleted tachyzoites shortly after invasion could be caused either by a defect in the formation or sealing of the parasitophorous vacuole membrane (PVM) during invasion, or by parasites activating egress shortly after internalisation is complete. To test if PKAc1 has a role in invasion, we measured the speed of invasion (S3A Fig) and the ability of invading tachyzoites to form a moving junction, as marked by rhoptry neck 4 (RON4) protein staining (S3B Fig). In both cases we saw no difference between parental and PKAc1-depleted lines (representative images shown of RON4 staining). We then determined whether PKAc1 plays a role in PVM formation. Secretion of rhoptries is required for PVM formation and can be measured by inhibiting invasion using cytochalasin D (CytD) and staining for the presence of ‘empty vacuoles’ (evacuoles) [39], using rhoptry protein 1 (ROP1) as a marker. Furthermore, we used microneme protein 8 (MIC8) cKD as a positive control, as this protein is known to be important in evacuole production [40]. Here, we observed that PKAc1-depleted tachyzoites were able to produce evacuoles at the same rate as parental lines, suggesting that this kinase plays no role in the secretion of rhoptry contents (S3C Fig). We also measured formation of the PVM on invading and invaded parasites using a mouse embryonic fibroblast (MEF) line that expresses membrane-bound tandem dimeric tomato (tdTomato) red fluorescent protein. [41]. In static images, in which PKAc1 and parental lines were expressing cytosolic GFP, we observed clear formation of an intense tdTomato^+^ membrane around the body of tachyzoites (S3Di Fig), as clearly demonstrated by plotting intensity values across a cross section (S3Dii Fig). Furthermore, when performing live cell imaging on tachyzoites invading tdTomato-expressing MEFs, we saw formation of a tdTomato^+^ membrane surrounding invading parasites, which persisted until collapse of the host cell (see S3E Fig and S6 Movie for representative time-lapse), further suggesting the formation of a PVM even in the absence of PKAc1.

We also assayed whether PKAc1 was involved in motility and host cell attachment, as well as microneme secretion (S4 Fig). To assay tachyzoite motility, we performed live cell imaging and quantitated total motile and nonmotile fractions as well as segregating based on the different types of motility when put in intracellular (IC) or extracellular (EC) buffer. Whilst we found an increase in the fraction of motile tachyzoites in EC buffer, we saw no changes upon repression of PKAc1 expression (S4Ai and S4Aii Fig). We also measured tachyzoite host cell attachment using standard assays and again found no differences of PKAc1-deficient parasites, as compared to controls (S4B Fig). Furthermore, we assayed secretion of micronemal proteins into the supernatant using standard conditions (in Ringer’s buffer), using both quantitative western blot and quantitative proteomics. In both cases, we found that PKAc1-depleted parasites had no difference to parental and no ATc controls (S4C, S4D and S4E Fig). Note that when performing quantitative proteomics on supernatants, we did see more peptides from proteins known or predicted to be found inside the parasite. Whilst we do not know the reasons behind this finding, we suggest that loss of PKAc1 may make the plasma membrane more fragile and prone to lysis (S4E Fig). Together, these results suggest that PKAc1 has no detectable role in invasion, motility, or host cell adhesion.

### PLP-1 is required for premature host cell egress in PKAc1-deficient tachyzoites

**Fig 4:**
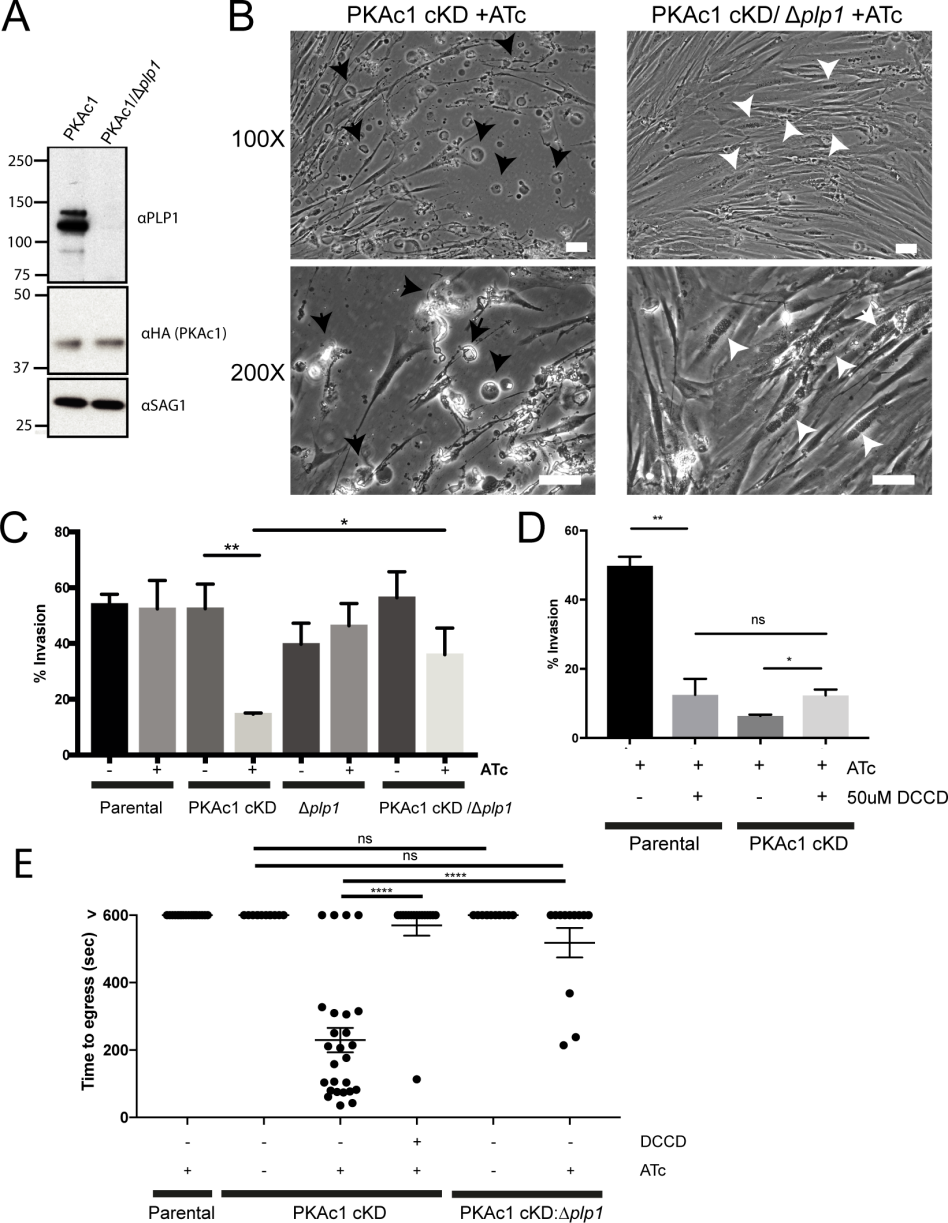
PLP-1 is required for host cell damage in PKAc1-deficient tachyzoites. (A) Genetic deletion of PLP-1 (Δ*plp1*) in PKAc1 cKD, as confirmed by western blot analysis. See S5 Fig for genetic strategy and genotyping. (B) Morphology of PKAc1 and PKAc1/PLP-1-deficient tachyzoites during in vitro growth in HFF after 24 hours of ATc treatment. White arrows point to intracellular tachyzoites after several rounds of replication. Black arrow point to examples of collapsed host cells. More images in S2 Fig. (C) Static invasion assay (after 10 minutes) of PKAc1 cKD/Δ*plp1* and Δ*plp1* tachyzoites as compared to PKAc1 cKD (reproduced from Fig 3A). (D) Effect of treatment with DCCD on host cell invasion of parental and PKAc1 cKD +ATc. Data from C and D represent mean ± SEM from three biological replicates. *P* values are calculated using an unpaired two-tailed *t* test, where * ≤ 0.05, ** ≤ 0.005, **** ≤ 0.00005, and ns = not significant. (E) Quantification of time to host cell collapse in Parental and PKAc1 cKD and PKAc1 cKD/Δ*plp1* tachyzoites ± ATc and ± 50 μM DCCD. Greater than 600 signifies that host cell collapse was not observed over the 10-minute (600-second) filming period. Parental + ATc, PKAc1 ± ATc data are reproduced from Fig 3 for purposes of comparison. Data represent mean ± 95% confidence intervals over at least three independent experiments, in which PKAc1 + ATc + 50 μM DCCD, *n* = 16; PKAc1/Δ*plp1* − ATc, *n* = 7; PKAc1/Δ*plp1* + ATc, *n* = 12. Individual numerical values underlying (C), (D), and (E) may be found in S1 Data. ATc, anhydrotetracycline; cKD, conditional knockdown; DCCD, N,N′-Dicyclohexylcarbodiimide; HA, haemagglutinin; HFF, human foreskin fibroblast; ns, not significant; PKAc1, protein kinase A catalytic subunit 1; PLP-1, perforin-like protein 1; SAG1, surface antigen 1.

We next determined whether PKAc1 was required for programmed host cell egress. *Toxoplasma* PLP-1 is critical to induce PVM and host cell membrane breakdown required for parasite egress [42,43]. Furthermore, vacuolar acidification is required for activating PLP-1 [23] to elicit membrane damage. We therefore wondered whether early egress of PKAc1-deficient tachyzoites requires acidification of the vacuolar space and the cytolytic activity of PLP-1. Furthermore, given that PLP-1 has no role in invasion [43], testing for dependency on PLP-1 and vacuolar acidification of the premature egress phenotype of PKAc1-depleted parasites would support a role for this kinase as a negative regulator of egress post-invasion. To test this, we first genetically deleted PLP-1 in our PKAc1 cKD line (S5 Fig). Western blot of PKAc1 cKD/Δ*plp-1* parasite lysates using PLP-1 antibodies showed a loss of signal verifying gene disruption (Fig 4A). To determine if PLP-1 is required for early egress of PKAc1-deficient parasites, we grew PKAc1 cKD and PKAc1 cKD/Δ*plp-1* tachyzoites in the presence of ATc for 24 hours and first monitored the morphology of parasite-infected host cells under the microscope. As previously shown in Fig 2, loss of PKAc1 results in collapsed host cells that appear to readily detach from the substrate, with no intracellular parasites evident (Fig 4B, black arrows). Conversely, host cells infected with parasites also lacking PLP-1 (PKAc1 cKD/Δ*plp-1* +ATc) appeared to have a reversal of this effect, containing many late-stage vacuoles full of tachyzoites, similar to parental and non-ATc-treated lines (Fig 4B, white arrows and S2 Fig). This suggests that loss of this cytolytic protein at least partially rescues genetic depletion of PKAc1. To quantify the role of PLP-1 in host cell destruction post-invasion in PKAc1-deficient tachyzoites, we performed an end-point invasion assay. As compared to the loss of PKAc1 alone (as reproduced from above), the additional deletion of PLP1 resulted in the presence of significantly more intracellular parasites at 10 minutes post-invasion (Fig 4C). As a control, we confirmed that loss of PLP-1 alone has no significant effect on invasion [43].

Previously, it has been shown that the inhibitor of H^+^ ATPase transporters N,N′-Dicyclohexylcarbodiimide (DCCD) can prevent vacuolar acidification and inhibit PLP-1 activity. We therefore also tested whether treatment of DCCD could reverse the loss of PKAc1, thus further supporting a role for this kinase in supressing egress post-invasion. Using a standard invasion assay, we observed that whilst DCCD treatment can inhibit invasion, it also mildly rescues the proportion of observable intracellular parasites of PKAc1-depleted tachyzoites after 10 minutes of invasion (Fig 4D). Furthermore, there was no difference in the amount of intracellular tachyzoites with and without PKAc1 that were both treated with DCCD (Fig 4D). This suggests that a H^+^ ATPase activity could play a role in vacuolar acidification, which drives re-exit of PKAc1-depleted *Toxoplasma* from host cells. It is important to note that DCCD treatment is not very specific and is likely inhibiting other H^+^ ATPases (e.g., mitochondrial), which is affecting the rate of invasion.

To confirm these findings and further dissect the PKAc1-depletion phenotype dependency on PLP-1 and acidification, we performed live cell imaging on PKAc1 cKD/Δ*plp-1* tachyzoites and quantitated how long tachyzoites remained intracellular following invasion. As compared to PKAc1-deficient parasites, the additional loss of PLP1 saw the vast majority of parasites remain intracellular over the 10 minutes of filming (Fig 4E). Tracking of individual PKAc1-depleted tachyzoites treated with 50 μM of DCCD showed that almost all parasites remain intracellular after invasion over the 10-minute filming period, suggesting that this compound is able to reverse the loss of PKAc1 of those parasites that are able to invade. Together, these data suggest that PKAc1 has a role in negatively regulating host cell egress and demonstrate that PKAc1-dependent egress from host cells occurs in a PLP-1-dependent fashion.

### PKAc1 controls rapid dampening of cytosolic Ca^2+^ after invasion

**Fig 5:**
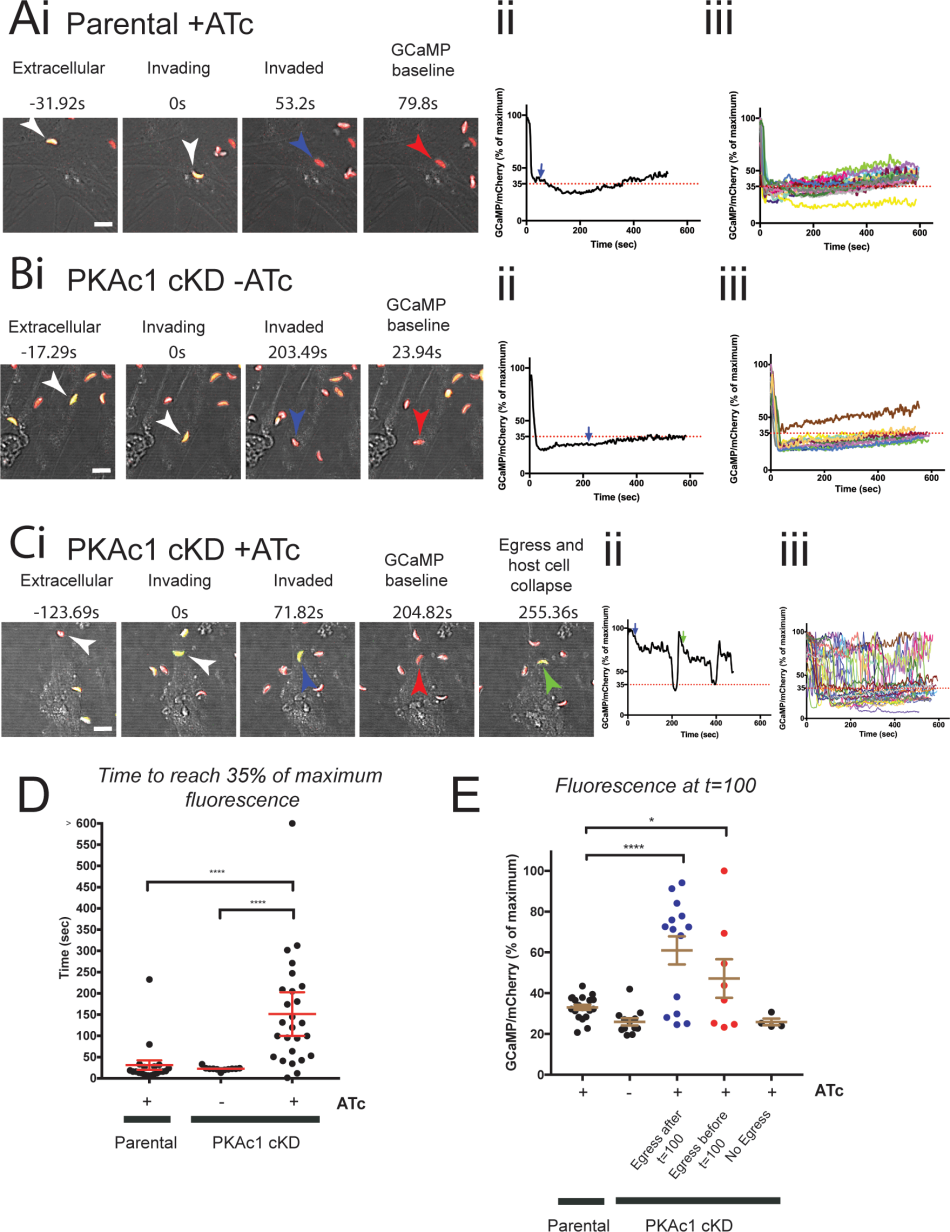
PKAc1 controls the rapid down-regulation of cytosolic Ca^2+^ shortly after invasion. (A) Live cell imaging of Parental tachyzoites with ATc treatment, (B) PKAc1 without ATc treatment, and (C) PKAc1 treated with ATc, all stably expressing GCaMP6f/mCherry at the *uprt* locus. In A, B, and C, (i) outlines images of individual movies, showing when parasites are extracellular, invading, and upon completion of invasion (blue arrow) at ‘baseline’ (defined as when GCaMP/mCherry ratio reaches 35%) (see accompanying S7, S8, and S9 Movies, respectively). In each case, (ii) shows a graphical representation of tracked parasites in (i), where blue line denotes the moment of completion of invasion and green arrow shows the moment of host cell egress. Dotted line marks 35% of maximum GCaMP/mCherry ratio, defined as ‘baseline’. In each case, (iii) shows overlays of all traced parasites. S6, S7 and S8 Figs show individual traces of each tracked parasite, respectively. (D) Graphical representation of each tracked parasite across all conditions showing time to ‘baseline’ fluorescence (defined as 35% of maximum). (E) Graphical representation of normalised GCaMP/mCherry ratio at *t* = 100 seconds across all parasites in all conditions, and in the case of PKAc1 cKD +ATc, segregated depending on time of egress. Data in D and E are represented as mean ± 95% confidence interval. *P* values are calculated pairwise, using an unpaired two-tailed *t* test, where * ≤ 0.05; **** ≤ 0.0001. Individual numerical values underlying (D) and (E) may be found in S1 Data. ATc, anhydrotetracycline; cKD, conditional knockdown; GCaMP, GFP-Calmodulin-M13-peptide-6; PKAc1, protein kinase A catalytic subunit 1.

Tachyzoite [Ca^2+^]_cyt_ has been temporally and functionally linked to parasite egress, motility, and invasion [11,12]. We hypothesised that PKAc1 may function to directly or indirectly negatively regulate [Ca^2+^]_cyt_, such that loss of this kinase sustains parasite motility and enables host cell egress to occur. Previously, we have used the genetically encoded biosensor, GFP-Calmodulin-M13-peptide-6 (GCaMP6), to monitor [Ca^2+^]_cyt_ in intracellular, egressing, and extracellular motile tachyzoites [11,44]. To first assess the utility of GCaMP6 in monitoring Ca^2+^ levels during invasion, we stably introduced the GCaMP6/mCherry-expressing plasmid at the *uprt* locus into the parental line (Δ*ku80*:TATi) and quantitated fluorescence levels (+ATc treatment) over a 10-minute period, acquiring five z-projections approximately every 1.3 seconds (Fig 5, S7 Movie). We observed that invasion of the parental line coincides with a rapid quenching of GCaMP6 fluorescence, dropping to 20%–40% of maximum levels in a period of 10–20 seconds post-invasion (*t* = 0 = beginning of invasion; blue arrow = completion of invasion; red arrow = time to reach 35% of maximum fluorescence) (Fig 5Ai and 5Aii, S7 Movie). We found that this pattern was seen in all observable invasion events in the parental line (Fig 5Aiii; for individual traces, see S6 Fig). We then introduced the GCaMP6/mCherry-expressing plasmid into PKAc1 cKD parasites and measured the dynamics in fluorescence as a readout for changes in [Ca^2+^]_cyt_. In the absence of ATc, PKAc1 cKD tachyzoites were able to quench [Ca^2+^]_cyt_ shortly after invasion, similar to the parental line (Fig 5Bi, 5Bii and 5Biii, and S8 Movie; individual traces are shown in S7 Fig). However, depletion of PKAc1 by ATc treatment resulted in drastic changes in [Ca^2+^]_cyt_ dynamics. Here, we noticed that PKAc1-depleted tachyzoites could not rapidly dampen GCaMP6 fluorescence after invasion was complete (green arrow = time of host cell collapse) (Fig 5Ci and 5Cii). Instead, as viewed by individual traces of PKAc1-depleted tachyzoites, [Ca^2+^]_cyt_ varied greatly during this time (Fig 5Ciii and S8 Fig). In order to quantitate and graphically represent cytosolic Ca^2+^ dynamics over time, we chose, based on parental controls, a value of 35% of maximum fluorescence to baseline after the completion of invasion (Fig 5Aii, 5Aiii, 5Bii and 5Biii). By measuring the time taken for tachyzoites to reach 35% of maximum, we could observe that PKAc1-depleted parasites took significantly longer than untreated or parental controls to reduce [Ca^2+^]_cyt_, averaging about 150 seconds to reach 35%, as compared to controls, which typically took 20–40 seconds (Fig 5D). We then measured GCaMP6 fluorescence level at 100 seconds, a time when all tachyzoites of control samples have completed invasion and dampened [Ca^2+^]_cyt_ (Fig 5A and 5B). Tachyzoites lacking PKAc1 expression were then split into those that egressed before and after *t* = 100, as well as those that did not egress at all. In doing so, we could see that those PKAc1-deficient parasites that did egress had significantly higher GCaMP6 fluorescence at this time point, whilst those that did not had levels equivalent to controls (Fig 5E). Overall, this work suggests that PKAc1 plays an important role in the rapid reduction of [Ca^2+^]_cyt_ that normally takes place shortly after invasion is complete.

### Live cell imaging reveals that PKAc1 regulates cytosolic [Ca^2+^] independent of PLP-1 activity

**Fig 6:**
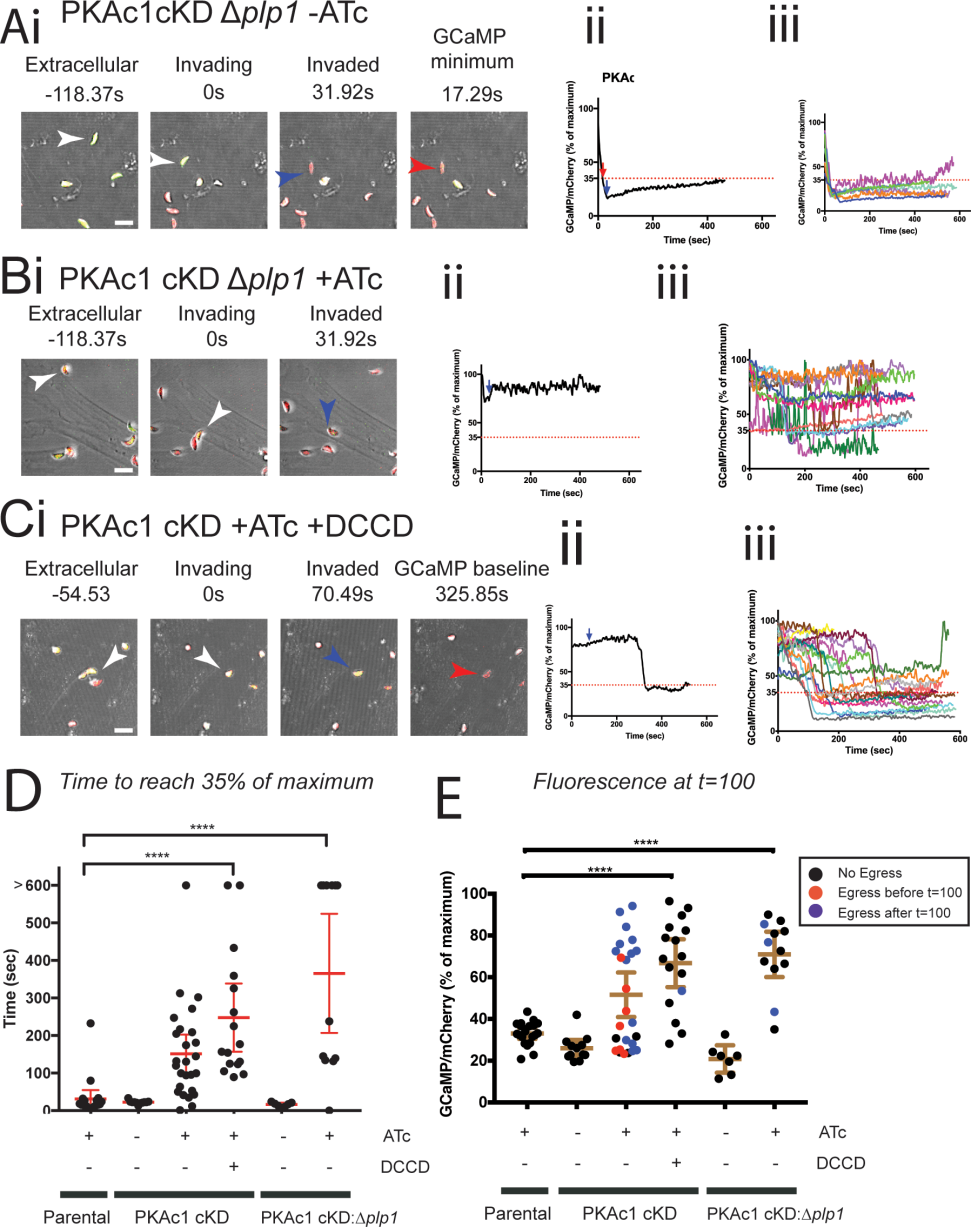
PLP-1 activity does not affect defective Ca^2+^ dynamics upon loss of PKAc1. (A) Live cell imaging of PKAc1 cKD/Δ*plp1* without and (B) with ATc. (C) PKAc1 cKD treated with ATc and 50 μM DCCD. All parasite lines are stably expressing GCaMP6/mCherry at the *uprt* locus. In A, B, and C, (i) shows images of the individual representative movie, showing stills when parasites are extracellular, invading, and upon completion of invasion (blue arrow) and then when the normalised GCaMP/mCherry ratio reaches 35% of maximum (see S10, S11 and S12 Movies, respectively). (ii) Shows, in each case, a graphical representation of tracked parasites in (i), where blue arrow denotes the moment of completion of invasion and green arrow shows the moment of host cell egress. Dotted line marks 35% of maximum GCaMP/mCherry ratio, defined as ‘baseline’. (iii) Shows, in each case, overlays of all traced parasites. S9 Fig shows individual traces of each tracked parasite for PKAc1 cKD/Δ*plp1*, with and without ATc, and S10 Fig outlines individual traces of PKAc1 cKD + ATc + 50 uM DCCD. (D) Graphical representation of each tracked parasite across all conditions, showing time to reach 35% of maximum, which is defined as ‘baseline’. Parental and PKAc1 cKD lines are from Fig 5. (E) Graphical representation of normalised GCaMP6/mCherry ratio at *t* = 100 seconds across all parasites in all conditions. Data in D and E are represented as mean ± 95% confidence interval. *P* values are calculated pairwise, using an unpaired two-tailed *t* test, where **** ≤ 0.0001. Individual numerical values underlying (D) and (E) may be found in S1 Data. ATc, anhydrotetracycline; cKD, conditional knockdown; DCCD, N,N′-Dicyclohexylcarbodiimide; GCaMP, GFP-Calmodulin-M13-peptide-6; PKAc1, protein kinase A catalytic subunit 1; PLP-1, perforin-like protein 1.

We wondered whether [Ca^2+^]_cyt_ in PKAc1-deficient parasites was influenced by PLP-1-dependent egress and exposure to the extracellular environment. To test this, we stably introduced GCaMP6/mCherry-expressing plasmid into the *uprt* locus of PKAc1 cKD/Δ*plp*1 tachyzoites (Fig 6). In the absence of ATc, PKAc1 cKD/Δ*plp1* tachyzoites invaded host cells in a typical fashion, which was followed by rapid dampening of [Ca^2+^]_cyt_, indistinguishable from parental controls (Fig 6Ai and 6Aii), which was highly consistent across the population (Fig 6Aiii). Upon depletion of PKAc1 with ATc, we saw that GCaMP6 levels did not dampen in a typical fashion, demonstrating that the cytolytic activity of PLP-1 and thus early exposure to the extracellular environment play little role in the loss of [Ca^2+^]_cyt_ dampening in PKAc1-deficient parasites (Fig 6Bi, 6Bii and 6Biii). To further explore the independence of [Ca^2+^]_cyt_ from membrane disruption by PLP-1, we treated parasites with the H^+^ ATPase inhibitor DCCD (Fig 6C). After tracking many invading tachyzoites, we saw that DCCD treatment of PKAc1-deficient parasites largely phenocopied PLP-1 deletion in terms of loss of egress, but sustained high [Ca^2+^]_cyt_ (Fig 6Ci, 6Cii and 6Ciii), thus further supporting the notion that regulation of [Ca^2+^]_cyt_ is not influenced by cytolytic activity of PLP-1. We also quantitated these effects by graphing time taken of each treatment to reach 35% of maximum fluorescence (Fig 6D), as well as fluorescence levels at *t* = 100 seconds (Fig 6E). As compared to PKAc1 depletion alone (redisplayed here from Fig 5), this showed that PLP-1-deficient and DCCD-treated PKAc1-deficient tachyzoites maintain a higher [Ca^2+^]_cyt_ level post-invasion (Fig 6D and 6E). Together, these data provide strong evidence that PKAc1 negatively regulates [Ca^2+^]_cyt_ post-invasion, independent of PLP-1-dependent PVM and host cell lysis.

### PKAc1 negatively regulates the basal cytosolic Ca^2+^ levels

**Fig 7:**
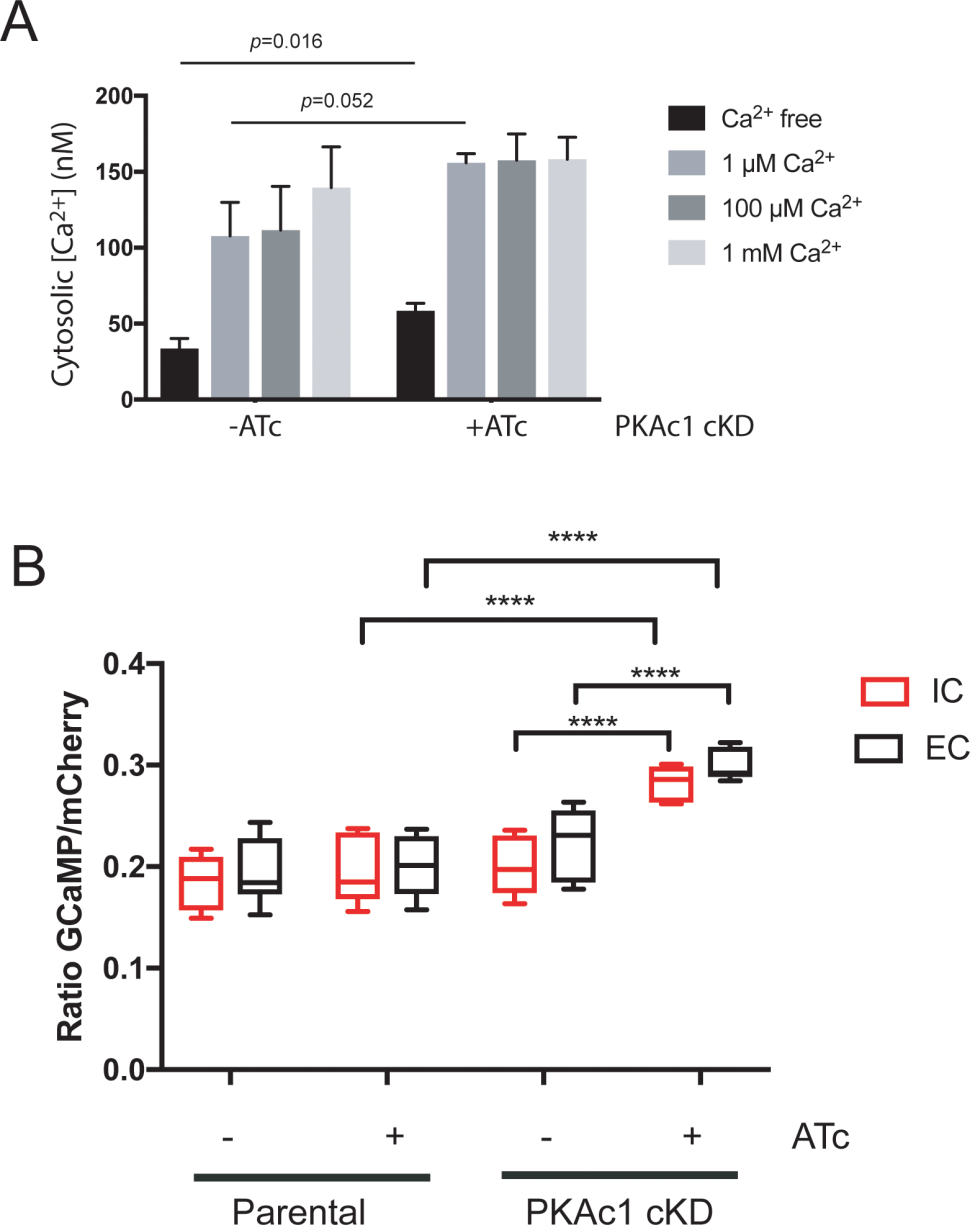
Cytosolic [Ca^2+^] are negatively regulated by PKAc1 in extracellular tachyzoites. (A) Measurements of intracellular Ca^2+^ using Fura-2 upon increasing concentration of extracellular Ca^2+^. Data represent mean ± SEM; statistical significance was assessed using unpaired *t* test. (B) FACS-based quantitation of GCaMP6/mCherry mean fluorescent intensity of Parental and PKAc1 cKD ± ATc in both IC and EC buffers. Values are from at least three independent replicates and are represented using box and whisker plots. *P* values are calculated pairwise using a paired two-tailed *t* test, where **** ≤ 0.0001. Individual numerical values underlying (A) and (B) may be found in S1 Data. ATc, anhydrotetracycline; cKD, conditional knockdown; EC, extracellular; FACS, fluorescence activated cell sorting; GCaMP6, GFP-Calmodulin-M13-peptide-6; IC, intracellular; PKAc1, protein kinase A catalytic subunit 1.

To further dissect the role of PKAc1 in negatively regulating [Ca^2+^]_cyt_, we investigated how the extracellular environment affects cytosolic concentrations of Ca^2+^. First of all, we tested if PKAc1 controls Ca^2+^ transport across the plasma membrane. To test this, we loaded extracellular tachyzoites with the Ca^2+^-responsive dye Fura-2 and performed calibrated measurements on parasite populations using fluorometry. We found that, when suspended in a Ca^2+^-free buffer, PKAc1-depleted parasites had a statistically higher [Ca^2+^]_cyt_, containing nearly 2-fold higher levels (about 58 nM compared with about 33 nM) (Fig 7A). PKAc1-depleted parasites were also observed to have higher [Ca^2+^]_cyt_ than PKAc1-expressing parasites when suspended in salines containing [Ca^2+^] of 1 μM, 100 μM, and 1 mM; however, in these cases, statistical significance was not reached (Fig 7A).

We also developed a fluorescence-activated cell sorting (FACS)-based assay to measure [Ca^2+^]_cyt_ using GCaMP6/mCherry-expressing parasites. This allowed us to further probe the relationship between environmental cues and the role of PKAc1 in negatively regulating [Ca^2+^]_cyt_. To do this, we resuspended tachyzoites in buffers mimicking the extracellular (EC) and intracellular (IC) environments and tested the role of PKAc1 in regulating [Ca^2+^]_cyt_ in these conditions (Fig 7B). Here, we could show that PKAc1-deficient parasites, even in IC buffer, had a significantly higher GCaMP6/mCherry signal, signifying a higher [Ca^2+^]_cyt_ than either PKAc1 cKD −ATc or parental controls (Fig 7B), suggesting that this cAMP-dependent protein kinase is critical for supressing [Ca^2+^]_cyt_ in intracellular conditions.

We then used live cell imaging to specifically interrogate any change in [Ca^2+^]_cyt_ in motile parasites in the presence and absence of PKAc1. Quantitating both maximum and minimum fluorescent values in motile parasites, we could show that PKAc1-deficient parasites only had statistically significant differences in minimum fluorescent levels in IC buffer (S11 Fig). Overall, this analysis suggests that PKAc1 plays an important role in regulating the resting level of [Ca^2+^]_cyt_ in conditions that mimic both the extracellular and intracellular environments.

### Loss of PKAc1 leads to a greater sensitivity to cGMP pathway stimulation

**Fig 8:**
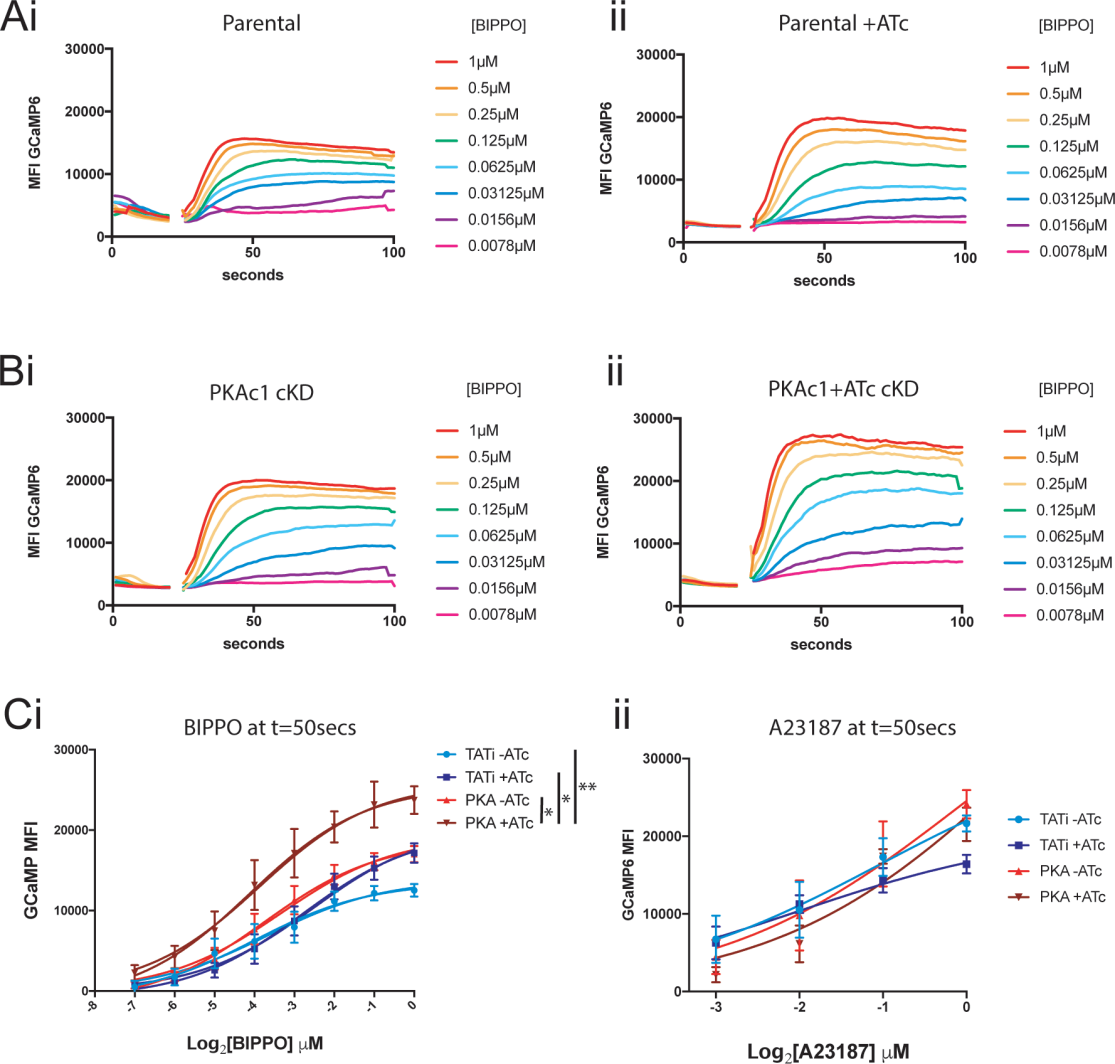
Loss of PKAc1 expression causes a greater sensitivity to cGMP-induced rise in cytosolic [Ca^2+^]. FACS-based analysis of GCaMP6-expressing (A) Parental and (B) PKAc1 cKD tachyzoites’ responses over time to a serial dilution of BIPPO in the absence (i) and presence (ii) of ATc. Data represent a single representative experiment. (C) Baseline-corrected dose–response curves at *t* = 50 seconds of GCaMP6-expressing Parental and PKAc1 cKD, ±ATc stimulated with either (i) BIPPO or (ii) A23187. Data represent mean ± SEM of at least three independent biological replicates. *P* values are calculated using a permutation test of the difference between two groups of growth curves. The *P* value is the proportion of permutations, where the mean *t* is greater in absolute value than the mean *t* for the original data set. Individual numerical values underlying (C) may be found in S1 Data. ATc, anhydrotetracycline; BIPPO, 5-benzyl-3-isopropyl-1H-pyrazolo[4,3-d]pyrimidin-7(6H)-one; cGMP, cyclic GMP; cKD, conditional knockdown; FACS, fluorescence-activated cell sorting; GCaMP6, GFP-Calmodulin-M13-peptide-6; MFI, mean fluorescent intensity; PKAc1, protein kinase A catalytic subunit 1; TATi, transactivator *Toxoplasma* inducible.

Previous work has shown that cGMP signalling positively regulates Ca^2+^ signalling in both *Toxoplasma* and *Plasmodium* spp. [11,45–47]. We therefore wondered whether PKAc1 controls [Ca^2+^]_cyt_ by regulating cGMP signalling. Furthermore, it has recently been shown that a putative cyclic nucleotide phosphodiesterase (PDE) in *Toxoplasma* has several PKAc1-dependent phosphorylation sites and a specific inhibitor ‘Compound 1’ of cGMP-dependent Protein kinase G (PKG) blocks premature egress in PKAc1 mutant parasites [48]. Therefore, it stands to reason that PKAc1 could act to control [Ca^2+^]_cyt_ by negatively regulating cGMP signalling. To test this, we developed a FACS-based assay to dynamically monitor [Ca^2+^]_cyt_ when cGMP signalling is activated, using the PDE inhibitor 5-benzyl-3-isopropyl-1H-pyrazolo[4,3-d]pyrimidin-7(6H)-one (BIPPO) [49]. We first measured basal GCaMP6 fluorescence in PKAc1 cKD and parental lines and then subjected them to a titration of [BIPPO] and measured fluorescence over time. In doing so, we were able to generate response curves over time and at a set time point (*t* = 50 seconds) (Fig 8A and 8B). Whilst we were able to show that the parental strain with or without ATc and PKAc1 cKD −ATc behaved similarly to a BIPPO titration, we found that PKAc1 depletion led to a greater rise in fluorescent levels at a given concentration. When subtracting background (to remove any effect of high resting [Ca^2+^]_cyt_ in PKAc1-depleted parasites) and plotted as a dose–response curve at *t* = 50 seconds over three independent experiments, it can be seen that loss of PKAc1 expression leads to a greater response (Fig 8Ci). Furthermore, we could show that this phenomenon is specific to use of BIPPO, as this is not seen when the same experiments were performed using the Ca^2+^ ionophore A23187 (Fig 8Cii). Overall, this work provides a functional link between PKAc1 and cGMP signalling to negatively regulate [Ca^2+^]_cyt_.

## Discussion

Critical to the establishment and perpetuation of infection by apicomplexan parasites is their ability to sense and respond to a changing host environment. Environmental cues allow for parasites to undergo differentiation when encountering a new host or to activate motility and egress at an optimal time during host cell infection cycles. Parasites must also switch off motility when invasion of a new host cell is complete in order to start replication. Several environmental factors have been identified that activate differentiation and motility in *Plasmodium* spp. and include blood lysophosphatidylcholine and mosquito xanthurenic acid, which activate sexual stage commitment and differentiation, respectively [50,51], whereas low extracellular [K^+^] [52,53] stimulates microneme secretion in blood and liver stages, and low pH (high [H^+^]) activates cell traversal in liver stages [54]. *Toxoplasma* too activates motility upon a drop in extracellular [K^+^] or pH, suggesting that sensing of some environmental cues is conserved across the Apicomplexa [22,23]. Despite the identification of these environmental cues, it is still not understood how apicomplexan parasites transduce these signals across the plasma membrane to induce Ca^2+^-dependent motility and differentiation.

cAMP/PKA signalling is commonly used to relay environmental changes and regulate cellular responses in eukaryotes. This typically occurs when GPCRs receive extracellular signals and activate ACs to produce cAMP or, alternatively, prevent its breakdown by inhibiting cAMP-specific 3′ 5′-PDEs. Despite apicomplexans having a striking paucity of GPCRs, we wanted to see if *Toxoplasma* uses cAMP/PKA signalling to relay environmental cues. Here, we identified a PKA orthologue—PKAc1—that localises to the parasite periphery, and performed immunoprecipitation of PKAc1 to identify interacting proteins. Using both gel- and whole eluate–based methods of protein identification, we could only robustly identify a likely regulatory subunit, PKAr1, suggesting that, unlike PKA in other eukaryotic systems, *Toxoplasma* PKAc1 does not stably interact with ACs, PDEs, or GPCRs. Furthermore, we demonstrate that PKAr1 likely derives membrane affinity through myristoylation and palmitoylation at its N-terminus, rather than through interactions with AKAP proteins, as is the case in metazoans. Together, this work suggests that PKAc1/PKAr1 either interacts with other proteins in much less stable complexes or that cAMP-dependent signalling in *Toxoplasma* is somewhat different from that in metazoans. In this regard, it is also interesting to note that some ACs (as well as guanylate cyclases) in Apicomplexa appear to be directly fused to ion transporter-like domains, suggesting a novel mechanism employed by this group of parasites to allow for sensing changes in extracellular ion concentrations and relaying these across the membrane to initiate intracellular signalling cascades [26,55].

Controlled depletion of PKAc1 highlighted the importance of this kinase during the *Toxoplasma* lytic cycle and revealed a unique phenotype, whereby tachyzoites rapidly exit the host cell shortly after invasion. In *P*. *falciparum*, PKA phosphorylates serine 610 (S610) on the cytoplasmic tail of the adhesin AMA1, and genetic mutation of this phospho-site leads to defects in invasion [25]. Given these findings in *P*. *falciparum* and the role that AMA1 plays in formation of the moving junction, we expected to find defects in invasion or sealing of the PVM in *Toxoplasma* PKAc1 mutants. However, using available assays and monitoring the formation of the PVM in real time using live cell imaging, we could not detect any apparent defects and instead could show that egress from host cells occurs well after invasion is complete, sometimes 200 seconds after. Supporting this notion, we demonstrated that loss of PLP-1 reverses loss of PKAc1 during host cell invasion, suggesting that aberrant activation of this cytolytic protein in this mutant is responsible for the phenotype. In saying this, the assays that we have available to us to monitor PVM biogenesis are not sensitive enough to determine if there is also a defect in sealing, and thus this remains a possibility that cannot be ruled out. It is also interesting to note that the cytoplasmic tail of AMA1 in *Toxoplasma* contains an aspartic acid at residue 557, the residue which is equivalent to S610 in *P*. *falciparum* [25]. Aspartic acids share physicochemical properties with phosphorylated serine residues, and thus *Toxoplasma* may have lost, or never gained, the capacity to regulate AMA1 tail function by PKA phosphorylation. It will be interesting in the future to compare the similarities and differences across Apicomplexa as to the function of cAMP signalling.

Our data suggest that PKAc1, upon invasion, negatively regulates cytosolic [Ca^2+^], allowing for the rapid suppression of the Ca^2+^ signal to promote a stable intracellular infection. This suggests that PKAc1-dependent signalling is important for sensing the host cell environment and suppressing [Ca^2+^]_cyt_, to turn off motility. [K^+^] is considered to be at a relatively high concentration inside host cells and lower in the extracellular environment, and previous reports have shown that [K^+^] acts as a major regulator of motility by somehow supressing [Ca^2+^]_cyt_. Using GCaMP6 as a [Ca^2+^]_cyt_ biosensor, we were also able to show that loss of PKAc1 in buffers mimicking the intracellular [K^+^] also resulted in aberrant [Ca^2+^]_cyt_. Together with our live cell imaging on GCaMP6-expressing parasites, our data suggest an important role of PKAc1 in negatively regulating [Ca^2+^]_cyt_ upon completion of invasion and sensing of the host cell environment.

Secretion of PLP-1 from the micronemes and its activation by PV acidification have both been shown to be important for host cell egress [23,42]. We have demonstrated that early egress by PKAc1-deficient tachyzoites is dependent on PLP-1 and PV acidification, but at this stage we have not been able to determine if PKAc1 directly regulates PLP-1 secretion and activity, or if they operate in different pathways that are both required for egress to take place. Given that PKAc1 negatively regulates [Ca^2+^]_cyt_ in a buffer that mimics the intracellular environment and that raised [Ca^2+^]_cyt_ levels are known to induce release of PLP-1 from the micronemes, it seems plausible that this is the reasonable mechanism that allows control of egress by cAMP signalling.

It was recently described that PKAc1 in *Toxoplasma* is also responsible for negatively regulating host cell egress [48]. Here, Jia and colleagues have used a conditional dominant negative PKAr1, which cannot respond to cAMP, to show that PKAc1 is important for negatively regulating early egress from host cells. Unfortunately, we were unable to robustly look at the role of PKAc1 during egress to confirm this work, because of the time it takes to deplete PKAc1 levels and the stochastic nature of host cell egress in standard *Toxoplasma* culturing conditions. However, our work is wholly consistent and complementary with their findings and furthermore also describes the role of PKAc1 in negatively regulating basal [Ca^2+^]_cyt_ levels, which appears especially important when encountering a host cell environment. Indeed, our findings provide a mechanism as to why dominant negative PKAr from Jia and colleagues can induce early egress and provide evidence as to why there is a preponderance of Ca^2+^-regulated proteins found to be more phosphorylated in the absence of PKAc1 activity [48]. Jia and colleagues also suggest that PKA and cGMP signalling are connected by identifying PKAc1-dependent phosphorylation sites on a putative PDE. We further this finding by showing that loss of PKAc1 sensitises parasites to cGMP-induced [Ca^2+^]_cyt_, thus providing a functional link between these three signalling modalities.

This work and the work of Jia and colleagues [48] suggest that cAMP signalling could be a mechanism by which *Toxoplasma* (and potentially other apicomplexan parasites) transduce extracellular signals. If this model is correct, then we would expect that cAMP levels would have a reciprocal relationship with [Ca^2+^]_cyt_, being high in intracellular parasites and low in extracellular parasites. Indeed, using available genetically encoded cAMP biosensors, this question is now eminently addressable and will be important to understand the role of cAMP in regulating motility. Interestingly, a recent study has demonstrated that another PKA orthologue, PKAc3, negatively regulates bradyzoite differentiation in *Toxoplasma*. Together with the results presented here and by Jia and colleagues, this is suggestive that a drop in cytosolic cAMP could have one of two outcomes: either egress from a host cell or differentiation into latent forms. Clearly, understanding more detail on the function of cAMP will be important if we wish to determine how *Toxoplasma* and other apicomplexan parasites appropriately respond to environmental cues to either exit the host cell or initiate differentiation.

## Materials and methods

### Plasmid construction

Detailed methodologies and oligonucleotides used for the construction of plasmids are available in the Supporting information (S1 Text and S2 Table, respectively).

### *Toxoplasma* transfection and in vitro culture

*T*. *gondii* tachyzoites were cultured under standard conditions. Briefly, HFFs (American Tissue culture collection [ATCC]), Vero, and immortalised MEFs expressing membrane-bound tdTomato [41] (a kind gift from C. Allison, WEHI) were grown in DME supplemented with 10% heat-inactivated Cosmic Calf Serum (Hyclone). When infecting HFFs with tachyzoites, media was exchanged to DME with 1% FCS. All cells were grown in humidified incubators at 37 °C/10% CO_2_. Transfection proceeded using either a Gene Pulser II (BioRad) or a Nucleofector 4D system (Lonza). Gene Pulser II transfection took place using standard procedures, using 50 μg of purified DNA, and the DNA was linearised if seeking homologous integration [56,57]. Nucleofection proceeded by using 2 × 10^6^ tachyzoites and 5 μg of DNA in 20 μL of buffer P3 (Lonza), which was pulsed using program F1-115.

### Plaque assays

Plaque assays were performed by inoculating 100–500 tachyzoites onto confluent monolayers and leaving the cells undisturbed for 7–8 days. Monolayers were then fixed in 80% ethanol and stained with crystal violet (Sigma).

### IFA

IFAs of *Toxoplasma*-infected host cells were undertaken using standard procedures detailed in S1 Text. Images were captured on an AP DeltaVision Elite microscope (GE Healthcare) equipped with a CoolSnap2 CCD detector and captured with SoftWorx software (GE Healthcare). Images were assembled using ImageJ and Adobe Illustrator software. Ty (mAb BB2), HA antibodies (3F10) (Roche), glydeosome-associated protein 45 (GAP45) [58], and ISP1 [30] were all used at 1:1,000 in 3% BSA/PBS. Secondary Alexa Fluor conjugated antibodies (Molecular Probes) were all used at 1:1,000. Images were adjusted in ImageJ and imported into Adobe Illustrator CC 2017.

### Host cell invasion assays

A detailed protocol for assaying invasion is available in S1 Text. The invasion assay proceeded largely as previously described [59]. Briefly, parasite lines were treated ±ATc and resuspended in IC buffer, to prevent invasion, and spun down onto the HFF monolayer in an eight-well chamber slide (Ibidi). IC buffer was exchanged for DME/1% FCS/10 mM HEPES, pH 7.5, and incubated for 10 minutes at 37 °C. Samples were then fixed, stained with anti-SAG1 mAb DG52 (1:3,000 dilution), permeabilised, and stained with rabbit anti-GAP45 (1:2,000) [58], followed by secondary antibodies, then imaging on a Zeiss Live Cell Observer and quantitation by manual counting. For invasion assays using GFP-expressing parasites, GAP45 staining was not carried out. Invasion assays with DCCD were carried out as described above except that parasites were allowed to invade in D1/HEPES medium containing 50 μM DCCD (Sigma). Graphs were generated and statistical tests performed using Prism 7 software.

### Host cell attachment assays

A detailed protocol for attachment assays is described in S1 Text. Tachyzoites were resuspended in DME/1% FCS/10 mM HEPES, pH 7.5, at a concentration of 1×10^7^ tachyzoites/mL. A total of 200 μL of tachyzoites was added to each well of an eight-well chamber slide (Ibidi) that contained HFFs fixed with 2.5% formaldehyde/0.02% glutaraldehyde. Parasites were allowed to attach for 30 minutes at 37 °C before fixing again and performing IFAs as described in S1 Text. Graphs were generated and statistical tests performed using Prism 7 software.

### Immunoprecipitation and proteomics

Egressed parasites from three T150 flasks for each of Δ*ku80*:TATi (parental) and Δ*ku80*:TATi:*PKAc1* cKD parasites were resuspended in 1 mL lysis buffer (100 mM HEPES, pH 7.5; 1% NP-40; 300 mM NaCl; 1 mM MgCl_2_; 25 U/mL Benzonase [Novagen], and protease inhibitors without EDTA [Roche]) and incubated on ice for 30 minutes, before centrifuging for 15 minutes at 14,000 rpm and 4 °C to pellet cellular debris. The lysates were added to 100 μL of anti-HA agarose beads (50% slurry [Sigma]) that had been washed twice with 1 mL PBS/0.5% NP-40) and incubated for 2.5 hours at 4 °C, with rotation. The beads were then pelleted by centrifugation at 10,000*g* for 15 seconds and the supernatants removed. The beads were washed five times with ice-cold 1 mL PBS/0.5% NP-40. Analysis was performed by running eluates on a NuPAGE 4%–12% nonreducing Bis-Tris Gel (Invitrogen) and stained with SyproRuby according to the manufacturer’s instructions (Invitrogen), followed by Page Blue staining (Fermentas). The two prominent bands from the Δ*ku80*:TATi:PKAc1 cKD IP were cut out and identified by mass spectrometry. Whole eluates were also subjected to mass spectrometry without prior protein gel separation and quantitated using a custom label-free quantification pipeline. For more details on procedures and stable isotope labelling by amino acids in cell culture (SILAC) labelling used for quantifying protein release from micronemes, please refer to S1 Text.

### Live cell imaging

Eight-well cell culture–treated chamber slides (Ibidi) were seeded with either HFFs or ROSA MEFs and grown as outlined above. *Toxoplasma* tachyzoites were treated overnight with 1 μg/mL of ATc and harvested as stated above. Tachyzoites were then resuspended in Endo buffer and spun down onto the host cells, then taken to the imaging station. Tachyzoites and host cells were then imaged using a Leica SP8 confocal, equipped with resonant scanner. For quantitative fluorescent measurements, GCaMP6 was excited with a 488-nm krypton/argon laser line, whilst mCherry and tdTomato were excited using the 594-nm and 561-nm laser lines, respectively. All imaging took place with pinhole at 1 Airy unit, typically over five z-stacks covering 5 μm. Image analysis took place using ImageJ, using inbuilt and custom macros (available on request). Data were processed in Excel and graphed in Prism.

### Fura-2–based intracellular Ca^2+^ measurements

PKAc1 cKD parasites grown in confluent HFFs for 24 hours were treated with 1 μg/mL ATc (or the equivalent volume of 100% EtOH; solvent control) for 6 hours. Following this, parasites were harvested by passage of cultures through a 26-gauge needle and centrifuged (1,500*g* for 10 minutes at 4 °C). The parasites were then resuspended in fresh culture medium containing either ATc (final concentration, 1 μg/mL) or the equivalent volume of 100% ethanol and added to flasks containing confluent HFFs for up to 24 hours. The parasites were then harvested from their host cells and loaded with Fura-2 as described previously [60]. Cytosolic Ca^2+^ measurements were performed at 37 °C using a PerkinElmer LS 50B Fluorescence Spectrometer as described previously [60]. Graphs were generated and statistical tests performed using Prism 7 software.

### FACS-based [Ca^2+^]_cyt_ measurements

Parental and PKAc1cKD parasites expressing GCaMP6/mCherry were treated ±ATc for 14 hours and resuspended in either EC buffer (141.8 mM NaCl, 5.8 mM KCl, 1 mM CaCl_2_, 1 mM MgCl_2_, 5.6 mM Glucose, 25 mM HEPES, pH 7.4) or IC buffer (5 mM NaCl, 142 mM KCl, 2 mM EGTA, 1 mM MgCl_2_, 5.6 mM Glucose, 25 mM HEPES, pH 7.4). Tachyzoite samples were then read on a FACS LSRII. Samples were spiked with 2× concentrated stocks of equivolume of either BIPPO or A23187 at 20 seconds post-acquisition and read for a total of 100 seconds. Values were imported into Prism 7 software for plotting and statistical testing.

### Microneme secretion assays

Secretion assays were performed and quantitated as described previously [61]. Briefly, cells were resuspended in Ringer’s buffer, shifted to 37 °C and allowed to secrete for 60 minutes. Secretion was arrested by placing cells on ice for 2 minutes. Parasites were separated from the soluble secreted proteins by centrifugation at 8,000 rpm, 4 °C, for 2 minutes. A total of 85 μL of supernatant was removed and centrifuged again to remove any remaining cells, and 75 μL of supernatant was removed and boiled with reducing Sample Buffer. The parasites pellet was washed with PBS and boiled in reducing Sample Buffer. Secreted proteins were analysed using antibodies to indicated factors by western blot: TOM40 (1:1,000) [62], GRA1 (1:2,000) [63], microneme protein 2 (MIC2) (1:5,000) [64], AMA1 CL22 (1:1,000) [65], SUB1(1:1,000) [66], and PLP1 [43].

## Supporting information

S1 Table Quantitative analysis of proteins secreted from extracellular tachyzoites. Parental and PKAc1 tachyzoites were grown in heavy and light SILAC media, respectively. Samples were mixed and subjected to tandem mass spectrometry (MS/MS) and peptides identified from proteins using Mascot and quantitated using a custom pipeline. Proteins highlighted in blue remain unchanged whilst those in green are statistically more abundant in PKAc1-depleted tachyzoites. MS/MS, tandem mass spectrometry; PKAc1, protein kinase A catalytic subunit 1; SILAC, stable isotope labelling by amino acids in cell culture.
(XLSX)

S2 Table Primers used in this study. (XLSX)

S1 Fig Generation of a tet-off conditional knockout of the *PKAc1* gene (TGME49_226030). Two gene flanks for HDR were designed to replace the endogenous *pkac1* promoter with the T7S4 ‘tet-off’ promoter. HDR fragments PCRed from genomic DNA and ligated into pPR2-HA_3_. The plasmid was linearised and transfected into Δ*ku80*:TATi and selected on Pyr to establish a stable population. Primers were designed to distinguish wild-type and genetically modified loci. Cloning strategy is outlined in S1 Text and primer sequences listed in S1 Table. Predicted sizes of resulting PCR products are listed adjacent to primers. (B) PCR on genomic DNA of parental and genetically modified parasites demonstrating predicted banding pattern of both parental and *pkac1* cKD genetically modified tachyzoites. cKD, conditional knockdown; HDR, homologous directed repair; pkac1, protein kinase A catalytic subunit 1; pPR2-HA_3_, plasmid for promoter replacement and HA-epitope tagging; Pyr, pyrimethamine.
(TIF)

S2 Fig Loss of PKAc1 leads to aberrant culture morphology. Images at 100× and 200× of different fields of view over three biological replicates, highlighting the morphology of host cells and the presence or absence of intracellular tachyzoites. White arrows highlight examples of intracellular parasites, both late stage and recently invaded. Black arrows highlight examples of damaged and dying host cells. Scale bar = 50 μm. PKAc1, protein kinase A catalytic subunit 1.
(TIF)

S3 Fig Loss of PKAc1 has no detectable effect on host cell invasion. (A) Speed of host cell invasion of parental and PKAc1-deficient tachyzoites, measured as time taken from point of attachment to complete invasion. Data represent mean ± SEM over 9–11 separate invasion events. *P* values are calculated using an unpaired two-tailed *t* test. (B) Representative visualisation of the moving junction formation as marked by RON4 antibodies pre-, mid-, and post-invasion in parental and PKAc1 cKD tachyzoites ±ATc treatment. (C) Evacuole formation of parental and PKAc1 tachyzoites ±ATc and MIC8 cKD (used as a positive control) [40]. Data represent mean ± SEM of three independent experiments. *P* values are calculated using an unpaired two-tailed *t* test, where * ≤ 0.05 and ns = not significant. (D) (i) Representative IFA and (ii) greyscale intensity plot of cross section (as denoted by white line) of a PKAc1 cKD/GFP +ATc having invaded a MEF host cell expressing membrane-bound tdTomato [41]. (E) Representative live cell imaging of PKAc1-deficient (+ATc) tachyzoites invading MEFs expressing membrane-bound tdTomato. White arrowheads track tachyzoite movement, whilst grey arrowheads denote accumulation of membrane-bound tdTomato around invading and intracellular tachyzoites. S6 Movie corresponds to this time series. See S7, S8, and S9 Movies for more examples. Individual numerical values underlying (A) and (C) may be found in S1 Data. ATc, anhydrotetracycline; cKD, conditional knockdown; evacuole, empty vacuole; GFP, green fluorescent protein; IFA, immunofluorescence assay; MEF, mouse embryotic fibroblast; MIC8 cKD, microneme protein 8 conditional knockdown; ns, not significant; PKAc1, protein kinase A catalytic subunit 1; RON4, rhoptry neck protein 4; tdTomato, tandem dimeric tomato red fluorescent protein.
(TIF)

S4 Fig Loss of PKAc1 has no detectable defect in motility, host cell attachment, or microneme secretion. (A) Two-dimensional quantitative motility assay of both parental and PKAc1 cKD ± ATc in either IC or EC buffer, in which the proportion of motile and nonmotile parasites were quantitated (i), as well as the type of motility (ii). (B) Quantitative host cell attachment assay of parental and PKAc1 cKD ±ATc normalised to Parental −ATc. (C) Representative microneme secretion assay using western blot showing secretion of a range of micronemal proteins between PKAc1 cKD ±ATc, comparing stimulation with A23187, BIPPO, or vehicle control (DMSO). (D) Graphical representation of quantitative analysis of MIC2 secretion by western blot and densitometry, when either stimulated with A23187 or vehicle control (DMSO) on PKAc1 cKD ±ATc. (E) Quantitative proteomic analysis of total secreted fraction of PKAc1 cKD +ATc versus Parental +ATc. Ratios were derived from averaging peptides across each protein and then plotted against the −log_10_ of their derived *P* value. Data presented in A, B, and D are mean ± SEM. *P* values are calculated using an unpaired two-tailed *t* test, where ns = not significant. Individual numerical values underlying (A), (B), and (D) may be found in S1 Data. ATc, anhydrotetracycline; BIPPO, 5-benzyl-3-isopropyl-1H-pyrazolo[4,3-d]pyrimidin-7(6H)-one; cKD, conditional knockdown; EC, extracellular; IC, intracellular; MIC2, microneme protein 2; PKAc1, protein kinase A catalytic subunit 1.
(TIF)

S5 Fig Generation of a PLP-1 knockout in PKAc1 cKD. (A) Genetic strategy of gene disruption. Two regions of homology, upstream and downstream of the PLP-1, were PCR amplified from genomic DNA and ligated either side of an expression cassette encoding CAT gene. Parasites were selected upon transfection of linearised plasmid and chloramphenicol treatment. Primers used for genotyping are shown and sequences listed in S1 Table. (B) Genotyping of parental and PKAc1 cKD:Δ*plp1* lines. Predicted sizes of PCR products using primers listed in ‘A’ are listed on the right. CAT, chloroamphenicol acetyl transferase; cKD, conditional knockdown; PKAc1, protein kinase A catalytic subunit 1; PLP-1, perforin-like protein 1.
(TIF)

S6 Fig Individual GCaMP6/mCherry intensity traces of Parental tachyzoites +ATc. Individual invading tachyzoites were tracked using ImageJ and an intensity ratio between GCaMP and mCherry was derived, followed by normalising against the maximum value. Thirty-five percent of maximum is marked with a dotted line to arbitrarily signify ‘baseline’ level. Blue arrow signifies moment of completed invasion. ATc, anhydrotetracycline; GCaMP6, GFP-Calmodulin-M13 peptide-6.
(TIF)

S7 Fig Individual GCaMP6/mCherry intensity traces of PKAc1 cKD tachyzoites without ATc treatment. Individual invading tachyzoites were tracked using ImageJ and an intensity ratio between GCaMP6 and mCherry was derived, followed by normalising against the maximum value. Thirty-five percent of maximum is marked with a dotted line to arbitrarily signify ‘baseline’ level. Blue arrow signifies moment of completed invasion. ATc, anhydrotetracycline; cKD, conditional knockdown; GCaMP6, GFP-Calmodulin-M13 peptide-6; PKAc1, protein kinase A catalytic subunit 1.
(TIF)

S8 Fig Individual GCaMP6/mCherry intensity traces of PKAc1 cKD tachyzoites with ATc treatment. Individual invading tachyzoites were tracked using ImageJ and an intensity ratio between GCaMP6 and mCherry was derived, followed by normalising against the maximum value. Thirty-five percent of maximum is marked with a dotted line to arbitrarily signify ‘baseline’ level. Blue arrow signifies moment of completed invasion. ATc, anhydrotetracycline; cKD, conditional knockdown; GCaMP6, GFP-Calmodulin-M13 peptide-6; PKAc1, protein kinase A catalytic subunit 1.
(TIF)

S9 Fig Individual GCaMP6/mCherry intensity traces of PKAc1 cKD/Δ*plp1* tachyzoites ±ATc treatment. Individual invading tachyzoites were tracked using ImageJ and an intensity ratio between GCaMP6 and mCherry was derived, followed by normalising against the maximum value. Thirty-five percent of maximum is marked with a dotted line to arbitrarily signify ‘baseline’ level. Blue arrow signifies moment of completed invasion and green arrows signifies moment of host cell egress. ATc, anhydrotetracycline; cKD, conditional knockdown; GCaMP6, GFP-Calmodulin-M13 peptide-6; PKAc1, protein kinase A catalytic subunit 1.
(TIF)

S10 Fig Individual GCaMP6/mCherry intensity traces of PKAc1 cKD tachyzoites + 50 μM DCCD. Individual invading tachyzoites were tracked using ImageJ and an intensity ratio between GCaMP6 and mCherry was derived, followed by normalising against the maximum value. Thirty-five percent of maximum is marked with a dotted line to arbitrarily signify ‘baseline’ level. Blue arrow signifies the moment of completed invasion and green arrows signify the moment of host cell egress. cKD, conditional knockdown; DCCD, N,N′-Dicyclohexylcarbodiimide; GCaMP6, GFP-Calmodulin-M13 peptide-6; PKAc1, protein kinase A catalytic subunit 1.
(TIF)

S11 Fig Quantitation of GCaMP6/mCherry-expressing parasites during motility. Parental and PKAc1 cKD parasites ±ATc tachyzoites expressing GCaMP6/mCherry were resuspended in either EC or IC buffer and allowed to glide on a glass coverslip. Motile parasites were tracked and maximum and minimum values extracted and compared across conditions. Data represent mean ± SD and *P* values were calculated by unpaired pairwise *t* tests. Individual numerical values underlying (i) and (ii) may be found in S1 Data. ATc, anhydrotetracycline; cKD, conditional knockdown; EC, extracellular; GCaMP6, GFP-Calmodulin-M13 peptide-6; IC, intracellular; PKAc1, protein kinase A catalytic subunit 1.
(PNG)

S1 Movie Live cell imaging of parental strain (Δ*ku80*/TATi) not treated with ATc. Slender arrow points to newly invaded tachyzoite (before filming commenced) and white arrow points to captured invasion event. Movie corresponds to still images represented in top panels of Fig 3B. ATc, anhydrotetracycline.
(AVI)

S2 Movie Live cell imaging of parental strain (Δ*ku80*/TATi) treated with ATc. White arrowhead points to tachyzoites undergoing invasion. Movie corresponds to still images represented in middle panels of Fig 3B. ATc, anhydrotetracycline.
(AVI)

S3 Movie Live cell imaging of PKAc1 cKD strain not treated with ATc. Tachyzoite captured invading in the centre of field of view. Movie corresponds to still images represented in bottom panels of Fig 3B. ATc, anhydrotetracycline; cKD, conditional knockdown; PKAc1, protein kinase A catalytic subunit 1.
(AVI)

S4 Movie Live cell imaging of PKAc1 cKD treated with ATc. Tachyzoite invasion precedes host cell collapse, followed by reactivation of motility. Movie corresponds to still images represented in top panels of Fig 3C. ATc, anhydrotetracycline; cKD, conditional knockdown; PKAc1, protein kinase A catalytic subunit 1.
(AVI)

S5 Movie Live cell imaging of PKAc1 cKD strain treated with ATc. Tachyzoite invasion precedes host cell collapse, followed by reactivation of motility. Movie corresponds to still images represented in bottom panels of Fig 3C. ATc, anhydrotetracycline; cKD, conditional knockdown; PKAc1, protein kinase A catalytic subunit 1.
(AVI)

S6 Movie Live cell imaging of PKAc1 cKD strain treated with ATc invading MEFs expressing membrane-bound tdtomato. Tdtomato signal represented in top two panels (single optical section and maximum projection) as greyscale images. Bottom panel represents transmitted light image of invading parasite. Movie corresponds to still images represented in bottom panels of S3 Fig panel E. ATc, anhydrotetracycline; cKD, conditional knockdown; MEF, mouse embryotic fibroblast; PKAc1, protein kinase A catalytic subunit 1; tdtomato, tandem dimeric tomato red fluorescent protein.
(AVI)

S7 Movie Live cell imaging of parental strain (Δ*ku80*/TATi) expressing GCaMP6/mCherry treated with ATc. An invading tachyzoite co-expressing GCaMP6 (green) and mCherry (red) is overlaid with transmitted light image. Movie corresponds to still images represented in panels of Fig 5Ai and GCaMP6/mCherry ratio graphed of Fig 5Aii. ATc, anhydrotetracycline; GCaMP6, GFP-Calmodulin-M13-peptide-6.
(AVI)

S8 Movie Live cell imaging of PKAc1 cKD strain expressing GCaMP6/mCherry not treated with ATc. An invading tachyzoite co-expressing GCaMP6 (green) and mCherry (red) is overlaid with transmitted light image. Movie corresponds to still images represented in panels of Fig 5Bi and GCaMP6/mCherry ratio graphed of Fig 5Bii. ATc, anhydrotetracycline; cKD, conditional knockdown; GCaMP6, GFP-Calmodulin-M13-peptide-6; PKAc1, protein kinase A catalytic subunit 1.
(AVI)

S9 Movie Live cell imaging of PKAc1 cKD strain expressing GCaMP6/mCherry treated with ATc. An invading tachyzoite co-expressing GCaMP6 (green) and mCherry (red) is overlaid with transmitted light image. Movie corresponds to still images represented in panels of Fig 5Ci and GCaMP6/mCherry ratio graphed of Fig 5Cii. ATc, anhydrotetracycline; cKD, conditional knockdown; GCaMP6, GFP-Calmodulin-M13-peptide-6; PKAc1, protein kinase A catalytic subunit 1.
(AVI)

S10 Movie Live cell imaging of PKAc1 cKD Δ*plp-1* strain expressing GCaMP6/mCherry not treated with ATc. An invading tachyzoite co-expressing GCaMP6 (green) and mCherry (red) is overlaid with transmitted light image. Movie corresponds to still images represented in panels of Fig 6Ai and GCaMP6/mCherry ratio graphed of Fig 6Aii. ATc, anhydrotetracycline; cKD, conditional knockdown; GCaMP6, GFP-Calmodulin-M13-peptide-6; PKAc1, protein kinase A catalytic subunit 1.
(AVI)

S11 Movie Live cell imaging of PKAc1 cKD Δ*plp-1* strain expressing GCaMP6/mCherry treated with ATc. An invading tachyzoite co-expressing GCaMP6 (green) and mCherry (red) is overlaid with transmitted light image. Movie corresponds to still images represented in panels of Fig 6Bi and GCaMP6/mCherry ratio graphed of Fig 6Bii. ATc, anhydrotetracycline; cKD, conditional knockdown; GCaMP6, GFP-Calmodulin-M13-6; PKAc1, protein kinase A catalytic subunit 1.
(AVI)

S12 Movie Live cell imaging of PKAc1 cKD Δ*plp-1* strain expressing GCaMP6/mCherry treated with ATc and DCCD. An invading tachyzoite co-expressing GCaMP6 (green) and mCherry (red) is overlaid with transmitted light image. Movie corresponds to still images represented in panels of Fig 6Ci and GCaMP6/mCherry ratio graphed of Fig 6Cii. ATc, anhydrotetracycline; cKD, conditional knockdown; DCCD, N,N′-Dicyclohexylcarbodiimide; GCaMP6, GFP-Calmodulin-M13-peptide-6; PKAc1, protein kinase A catalytic subunit 1.
(AVI)

S1 Data (XLSX)

S1 Text (DOCX)

## Abbreviations

AC: adenyl cyclase
AKAP: A-kinase-associated protein
AMA1: apical membrane antigen 1
ATc: anhydrotetracycline
cAMP: cyclic adenosine monophosphate
BIPPO: 5-benzyl-3-isopropyl-1H-pyrazolo[4,3-d]pyrimidin-7(6H)-one
[Ca^2+^]_cyt_: cytosolic concentration of Ca^2+^
CDPK: Ca^2+^-dependent protein kinase
cGMP: cyclic GMP
cKD: conditional knockdown
CRISPR: clustered regularly interspaced short palindromic repeats
CytD: cytochalasin D
DCCD: N,N′-Dicyclohexylcarbodiimide
DHFR: dihydrofolate reductase
evacuole: empty vacuole
FACS: fluorescence-activated cell sorting
GAC: glideosome-associated connector
GAP45: glydeosome-associated protein 45
GCaMP6: GFP-Calmodulin-M13-peptide-6
GFP: green fluorescent protein
GPCR: G-protein coupled receptor
[H^+^]: concentration of H^+^
HA: haemagglutinin epitope
HFF: human foreskin fibroblast
IFA: immunofluorescence assay
IMC: inner membrane complex
IMC1: inner membrane complex protein 1
ISP1: IMC sub-compartment protein 1
[K^+^]: concentration of K^+^
MEF: mouse embryonic fibroblast
MIC2: microneme protein 2
MIC8: microneme protein 8
MS/MS: tandem mass spectrometry
MyoA: Myosin A
PDE: phosphodiesterase
PKA: protein kinase A
PKAc: protein kinase A catalytic subunit
PKAc1: protein kinase A catalytic subunit 1
PKAr: protein kinase A regulatory subunit
PKAr1: protein kinase regulatory subunit 1
PKG: protein kinase G
PLP-1: perforin-like protein 1
PV: parasitophorous vacuole
PVM: parasitophorous vacuole membrane
RON4: rhoptry neck protein 4
ROP1: rhoptry protein 1
S610: serine 610
SILAC: stable isotope labelling by amino acids in cell culture
TATi: transactivator of Toxoplasma inducible
tdTomato: tandem dimeric tomato red fluorescent protein
tet-off: tetracycline-off
T7S4: tetO7sag4 promoter
WT: wild type
