# Supplementary figures and images for: Protein kinase A negatively regulates Ca2+ signalling in Toxoplasma gondii
Source: PLoS Biol. 2018 Sep 12;16(9):e2005642. doi: 10.1371/journal.pbio.2005642 (PMC6152992; doi:10.1371/journal.pbio.2005642)

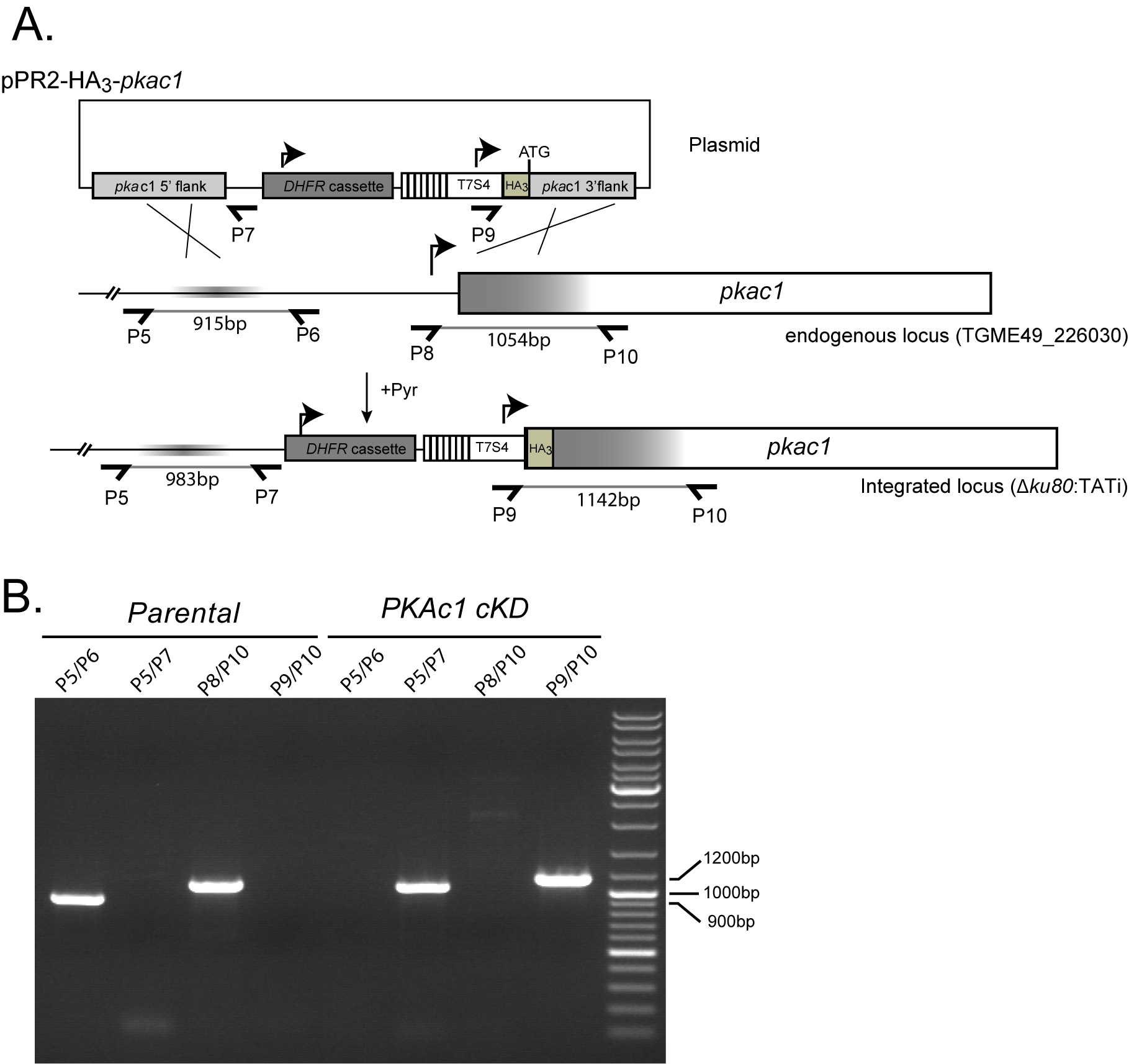

Supplement: S1 Fig — Two gene flanks for HDR were designed to replace the endogenous pkac1 promoter with the T7S4 ‘tet-off’ promoter. HDR fragments PCRed from genomic DNA and ligated into pPR2-HA3. The plasmid was linearised and transfected into Δku80:TATi and selected on Pyr to establish a stable population. Primers were designed to distinguish wild-type and genetically modified loci. Cloning strategy is outlined in S1 Text and primer sequences listed in S1 Table. Predicted sizes of resulting PCR products are listed adjacent to primers. (B) PCR on genomic DNA of parental and genetically modified parasites demonstrating predicted banding pattern of both parental and pkac1 cKD genetically modified tachyzoites. cKD, conditional knockdown; HDR, homologous directed repair; pkac1, protein kinase A catalytic subunit 1; pPR2-HA3, plasmid for promoter replacement and HA-epitope tagging; Pyr, pyrimethamine. (TIF) [file pbio.2005642.s003.tif]

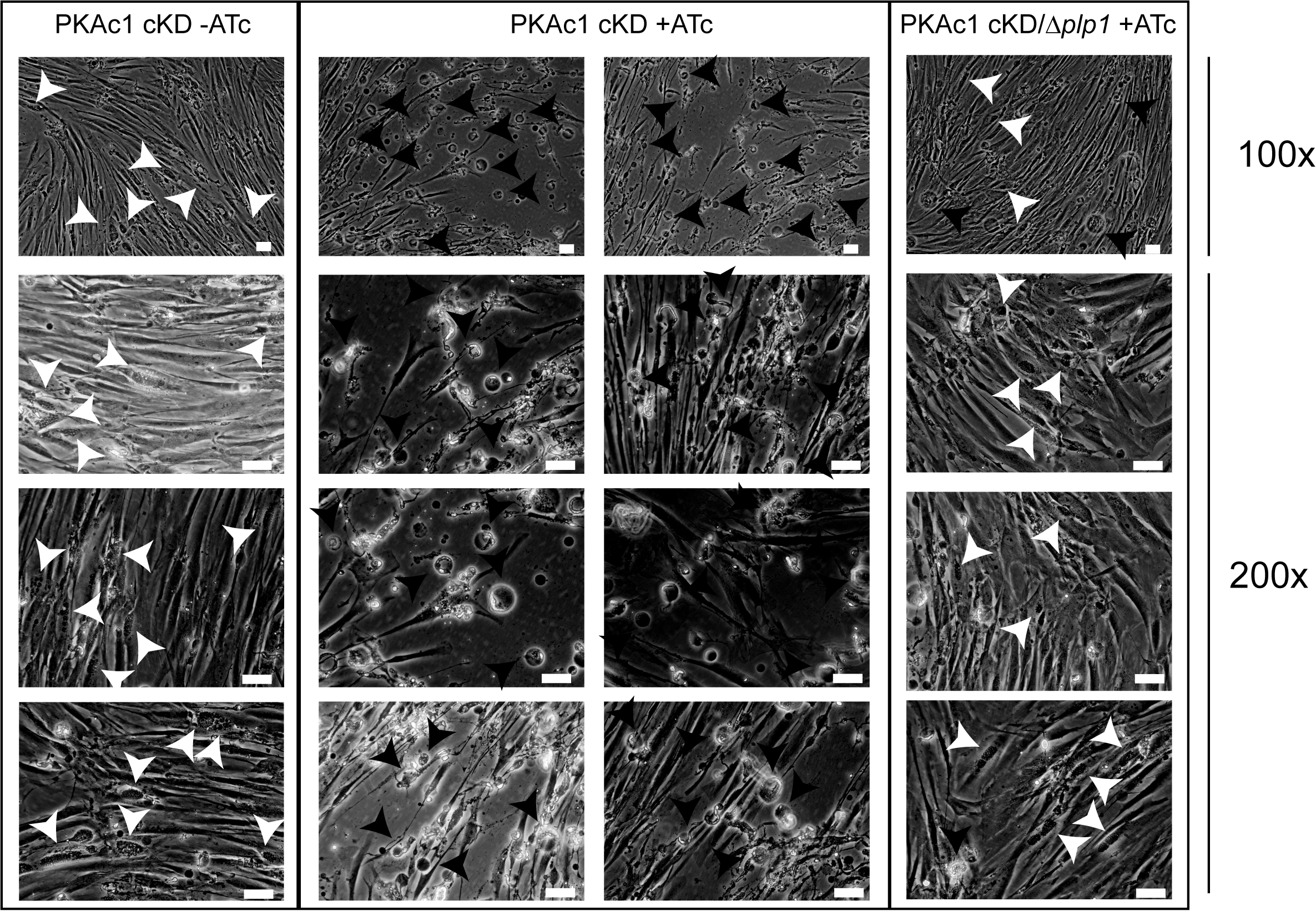

Supplement: S2 Fig — Images at 100× and 200× of different fields of view over three biological replicates, highlighting the morphology of host cells and the presence or absence of intracellular tachyzoites. White arrows highlight examples of intracellular parasites, both late stage and recently invaded. Black arrows highlight examples of damaged and dying host cells. Scale bar = 50 μm. PKAc1, protein kinase A catalytic subunit 1. (TIF) [file pbio.2005642.s004.tif]

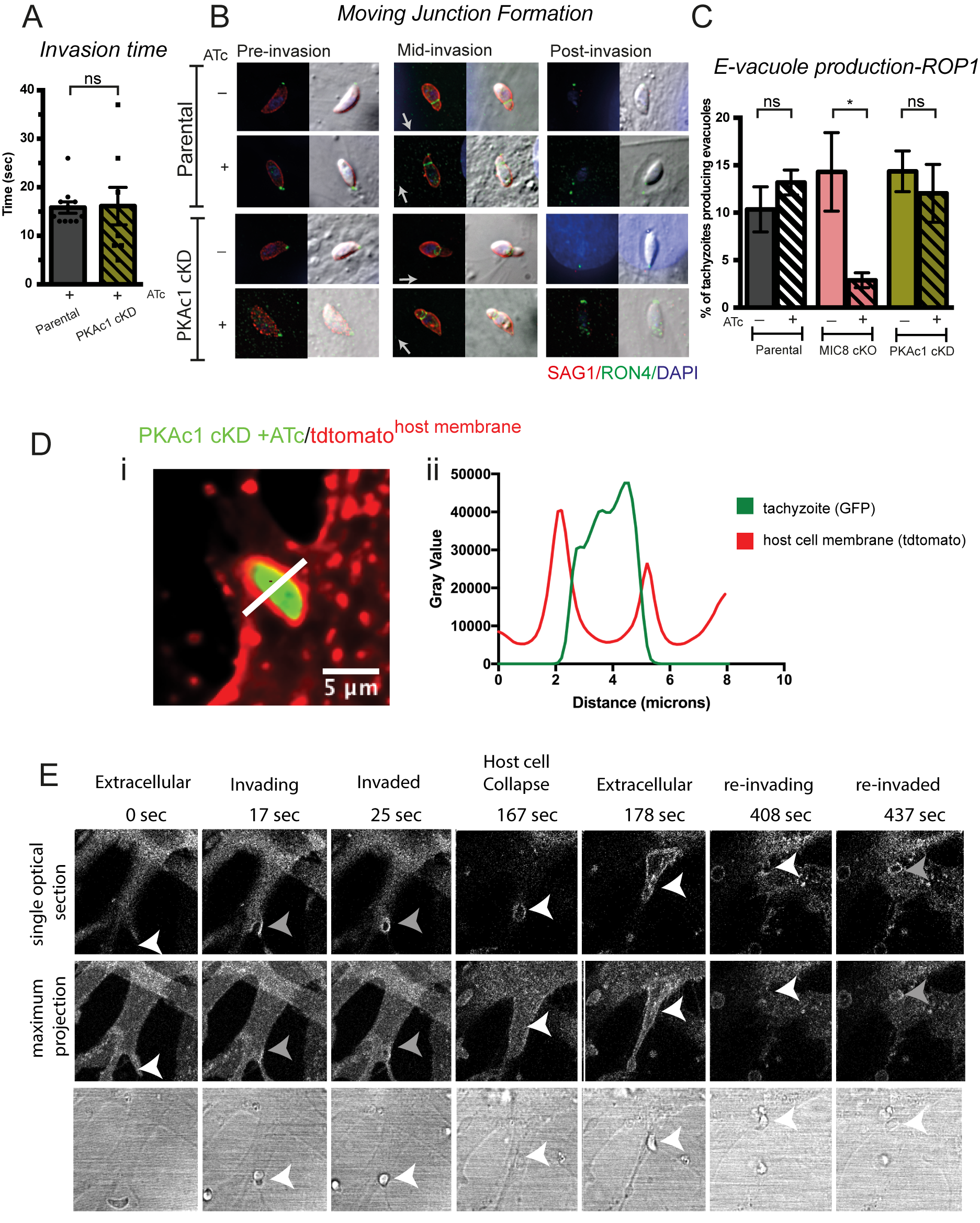

Supplement: S3 Fig — (A) Speed of host cell invasion of parental and PKAc1-deficient tachyzoites, measured as time taken from point of attachment to complete invasion. Data represent mean ± SEM over 9–11 separate invasion events. P values are calculated using an unpaired two-tailed t test. (B) Representative visualisation of the moving junction formation as marked by RON4 antibodies pre-, mid-, and post-invasion in parental and PKAc1 cKD tachyzoites ±ATc treatment. (C) Evacuole formation of parental and PKAc1 tachyzoites ±ATc and MIC8 cKD (used as a positive control) [40]. Data represent mean ± SEM of three independent experiments. P values are calculated using an unpaired two-tailed t test, where * ≤ 0.05 and ns = not significant. (D) (i) Representative IFA and (ii) greyscale intensity plot of cross section (as denoted by white line) of a PKAc1 cKD/GFP +ATc having invaded a MEF host cell expressing membrane-bound tdTomato [41]. (E) Representative live cell imaging of PKAc1-deficient (+ATc) tachyzoites invading MEFs expressing membrane-bound tdTomato. White arrowheads track tachyzoite movement, whilst grey arrowheads denote accumulation of membrane-bound tdTomato around invading and intracellular tachyzoites. S6 Movie corresponds to this time series. See S7, S8, and S9 Movies for more examples. Individual numerical values underlying (A) and (C) may be found in S1 Data. ATc, anhydrotetracycline; cKD, conditional knockdown; evacuole, empty vacuole; GFP, green fluorescent protein; IFA, immunofluorescence assay; MEF, mouse embryotic fibroblast; MIC8 cKD, microneme protein 8 conditional knockdown; ns, not significant; PKAc1, protein kinase A catalytic subunit 1; RON4, rhoptry neck protein 4; tdTomato, tandem dimeric tomato red fluorescent protein. (TIF) [file pbio.2005642.s005.tif]

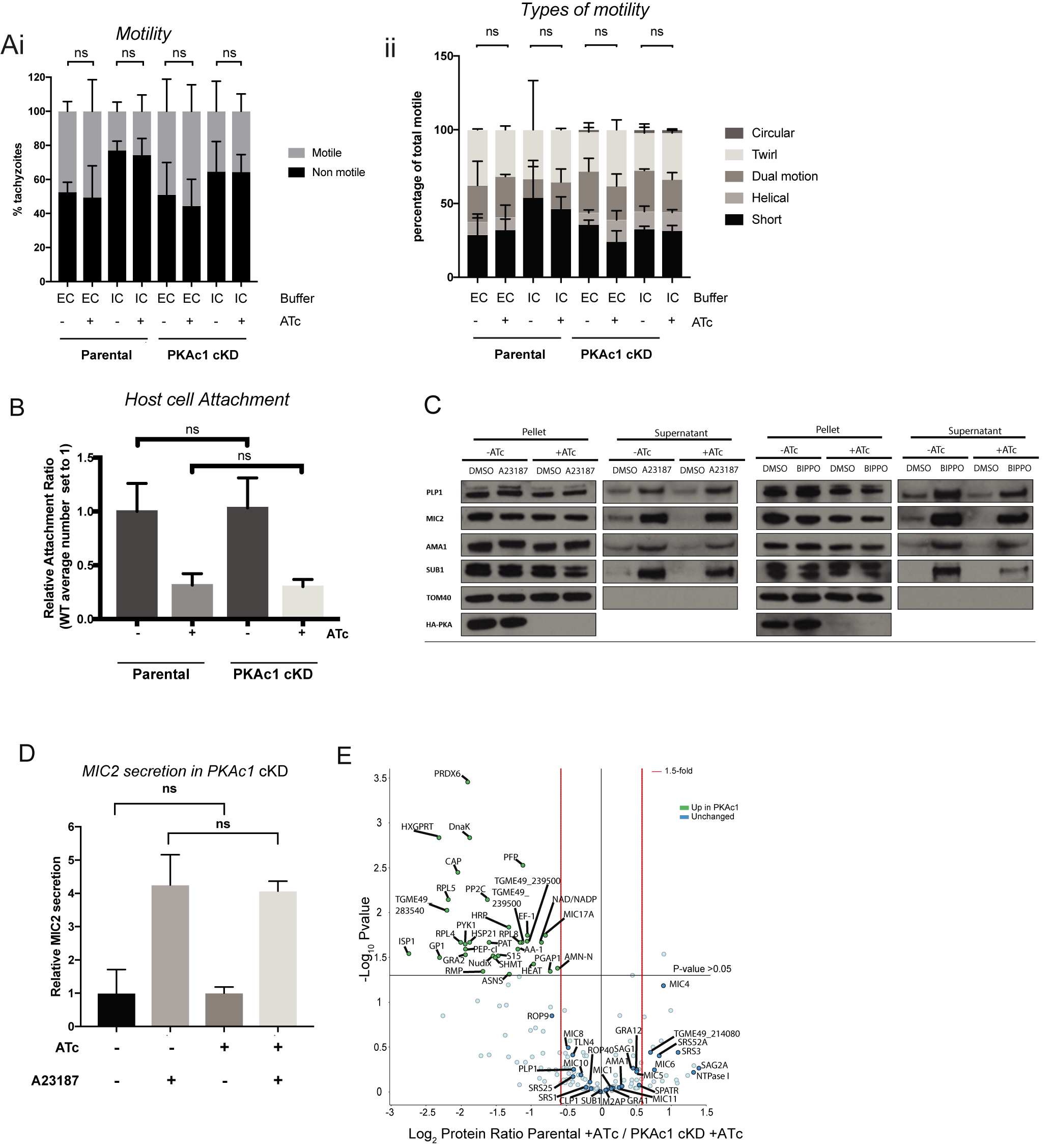

Supplement: S4 Fig — (A) Two-dimensional quantitative motility assay of both parental and PKAc1 cKD ± ATc in either IC or EC buffer, in which the proportion of motile and nonmotile parasites were quantitated (i), as well as the type of motility (ii). (B) Quantitative host cell attachment assay of parental and PKAc1 cKD ±ATc normalised to Parental −ATc. (C) Representative microneme secretion assay using western blot showing secretion of a range of micronemal proteins between PKAc1 cKD ±ATc, comparing stimulation with A23187, BIPPO, or vehicle control (DMSO). (D) Graphical representation of quantitative analysis of MIC2 secretion by western blot and densitometry, when either stimulated with A23187 or vehicle control (DMSO) on PKAc1 cKD ±ATc. (E) Quantitative proteomic analysis of total secreted fraction of PKAc1 cKD +ATc versus Parental +ATc. Ratios were derived from averaging peptides across each protein and then plotted against the −log10 of their derived P value. Data presented in A, B, and D are mean ± SEM. P values are calculated using an unpaired two-tailed t test, where ns = not significant. Individual numerical values underlying (A), (B), and (D) may be found in S1 Data. ATc, anhydrotetracycline; BIPPO, 5-benzyl-3-isopropyl-1H-pyrazolo[4,3-d]pyrimidin-7(6H)-one; cKD, conditional knockdown; EC, extracellular; IC, intracellular; MIC2, microneme protein 2; PKAc1, protein kinase A catalytic subunit 1. (TIF) [file pbio.2005642.s006.tif]

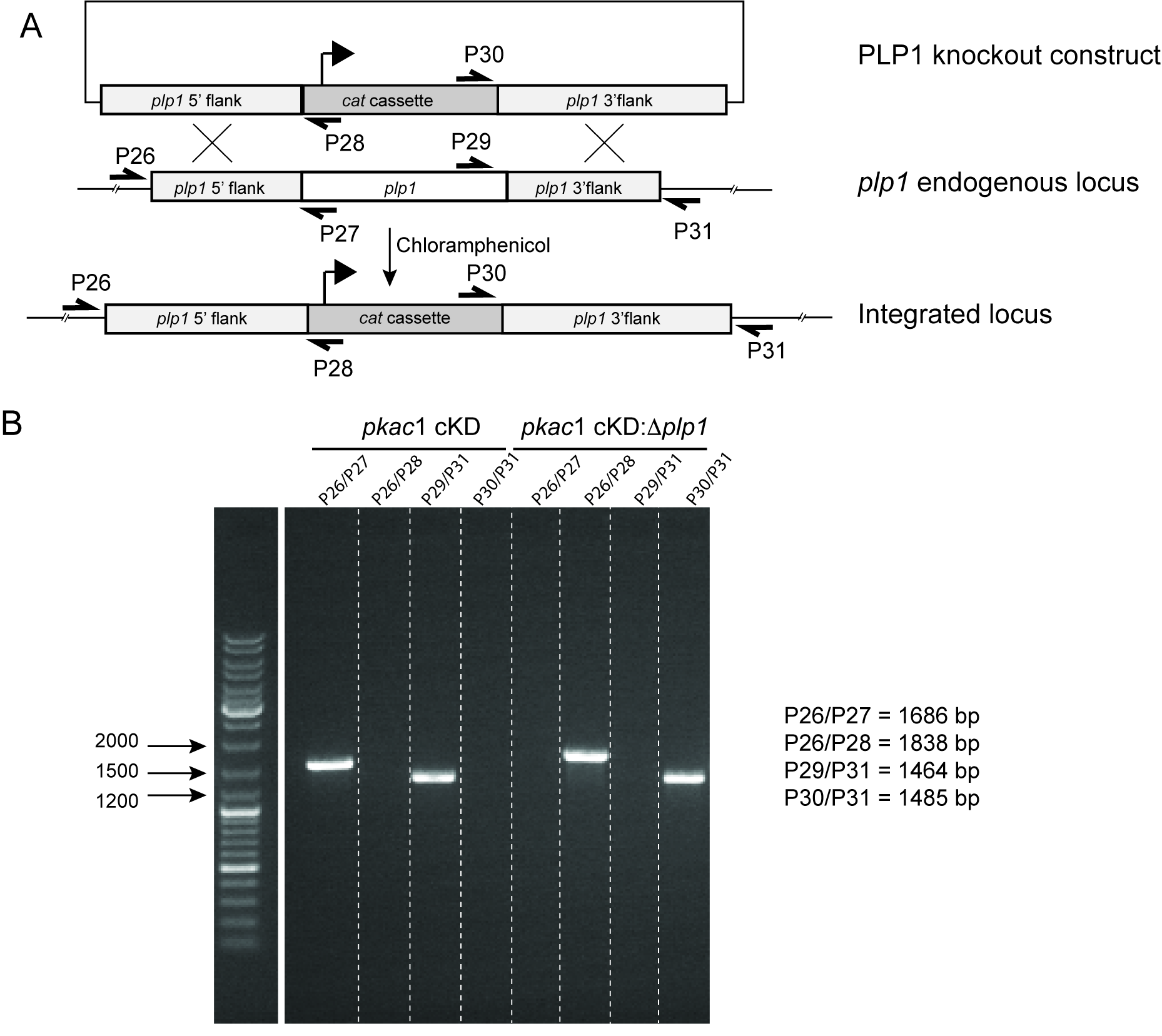

Supplement: S5 Fig — (A) Genetic strategy of gene disruption. Two regions of homology, upstream and downstream of the PLP-1, were PCR amplified from genomic DNA and ligated either side of an expression cassette encoding CAT gene. Parasites were selected upon transfection of linearised plasmid and chloramphenicol treatment. Primers used for genotyping are shown and sequences listed in S1 Table. (B) Genotyping of parental and PKAc1 cKD:Δplp1 lines. Predicted sizes of PCR products using primers listed in ‘A’ are listed on the right. CAT, chloroamphenicol acetyl transferase; cKD, conditional knockdown; PKAc1, protein kinase A catalytic subunit 1; PLP-1, perforin-like protein 1. (TIF) [file pbio.2005642.s007.tif]

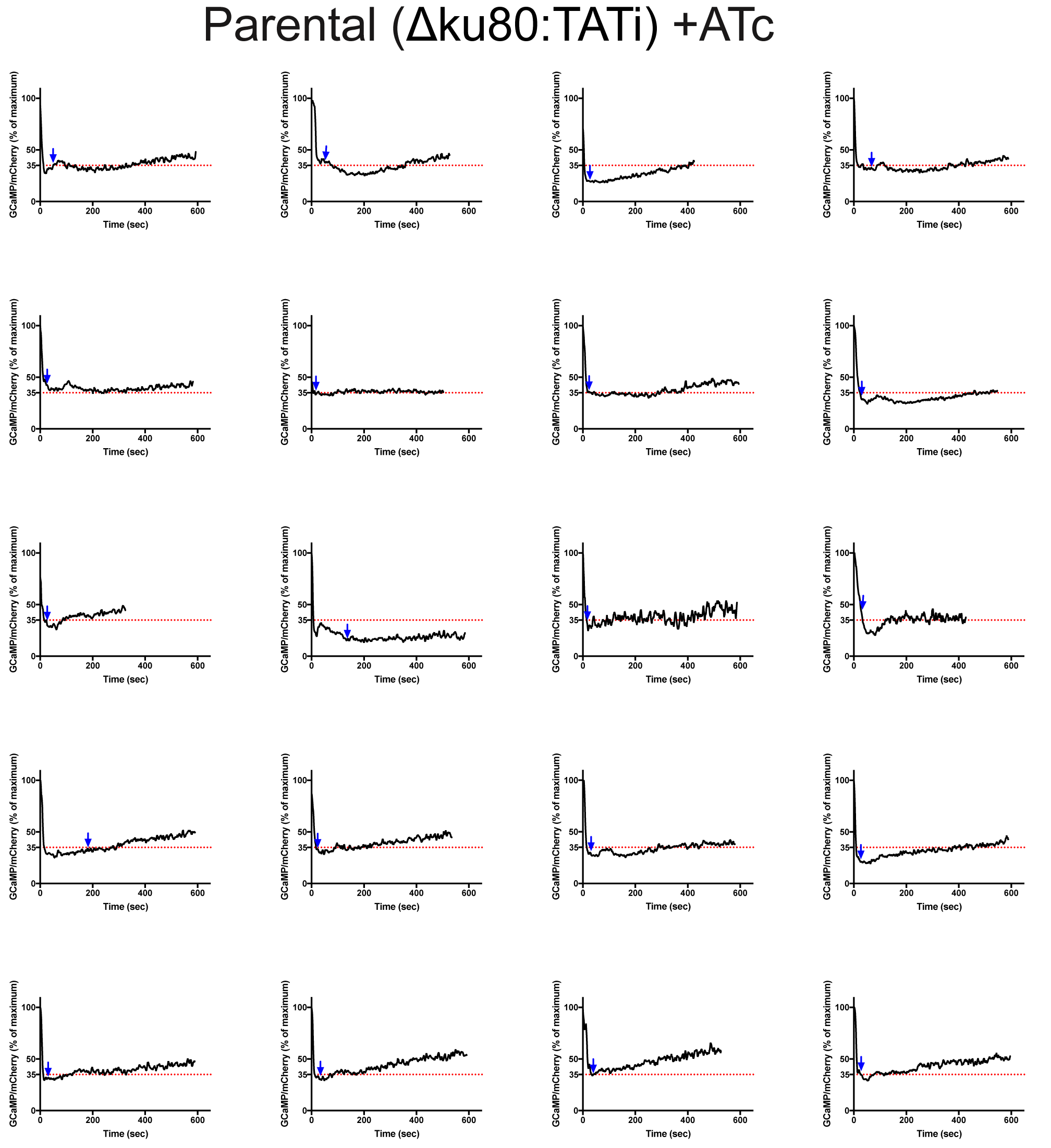

Supplement: S6 Fig — Individual invading tachyzoites were tracked using ImageJ and an intensity ratio between GCaMP and mCherry was derived, followed by normalising against the maximum value. Thirty-five percent of maximum is marked with a dotted line to arbitrarily signify ‘baseline’ level. Blue arrow signifies moment of completed invasion. ATc, anhydrotetracycline; GCaMP6, GFP-Calmodulin-M13 peptide-6. (TIF) [file pbio.2005642.s008.tif]

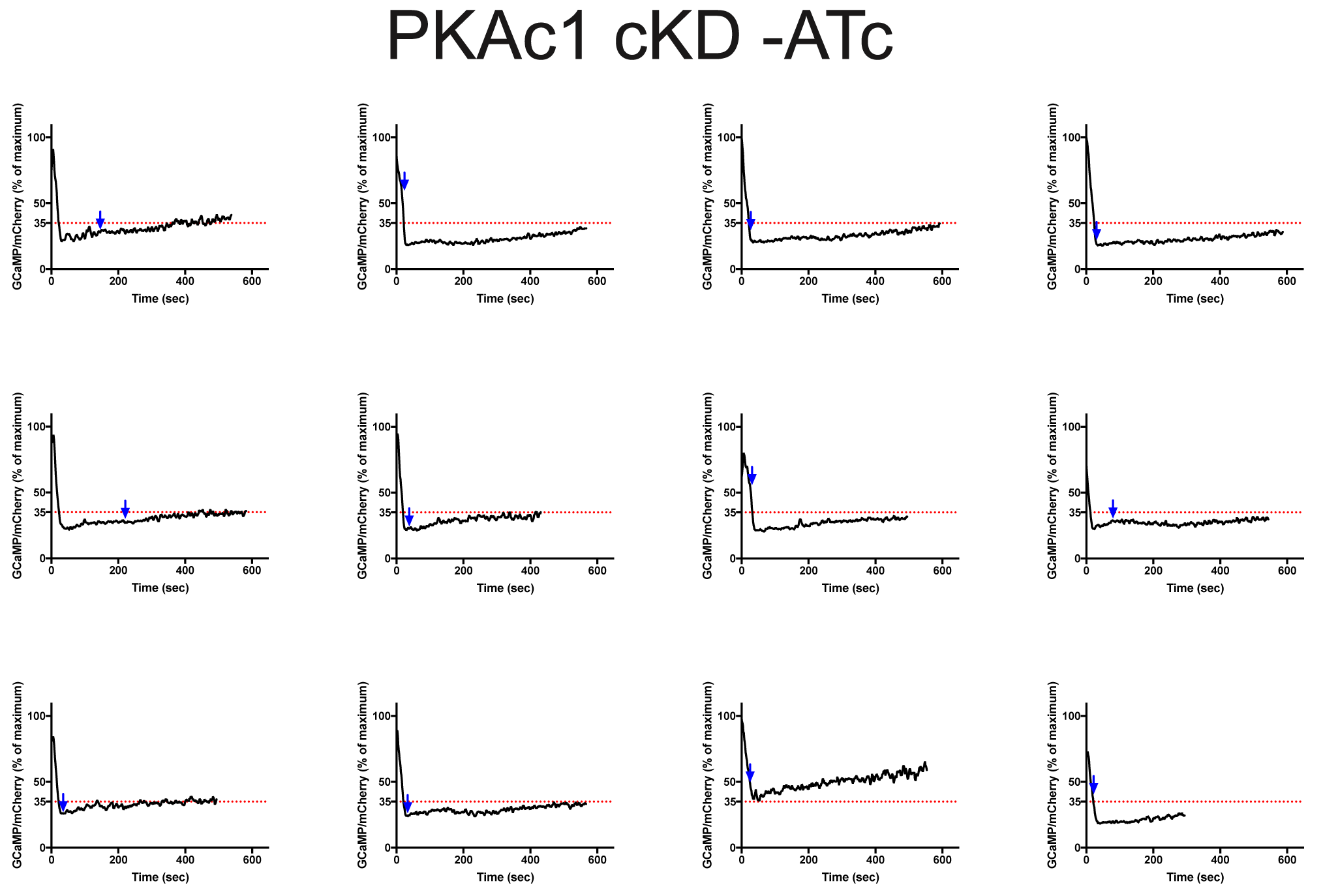

Supplement: S7 Fig — Individual invading tachyzoites were tracked using ImageJ and an intensity ratio between GCaMP6 and mCherry was derived, followed by normalising against the maximum value. Thirty-five percent of maximum is marked with a dotted line to arbitrarily signify ‘baseline’ level. Blue arrow signifies moment of completed invasion. ATc, anhydrotetracycline; cKD, conditional knockdown; GCaMP6, GFP-Calmodulin-M13 peptide-6; PKAc1, protein kinase A catalytic subunit 1. (TIF) [file pbio.2005642.s009.tif]

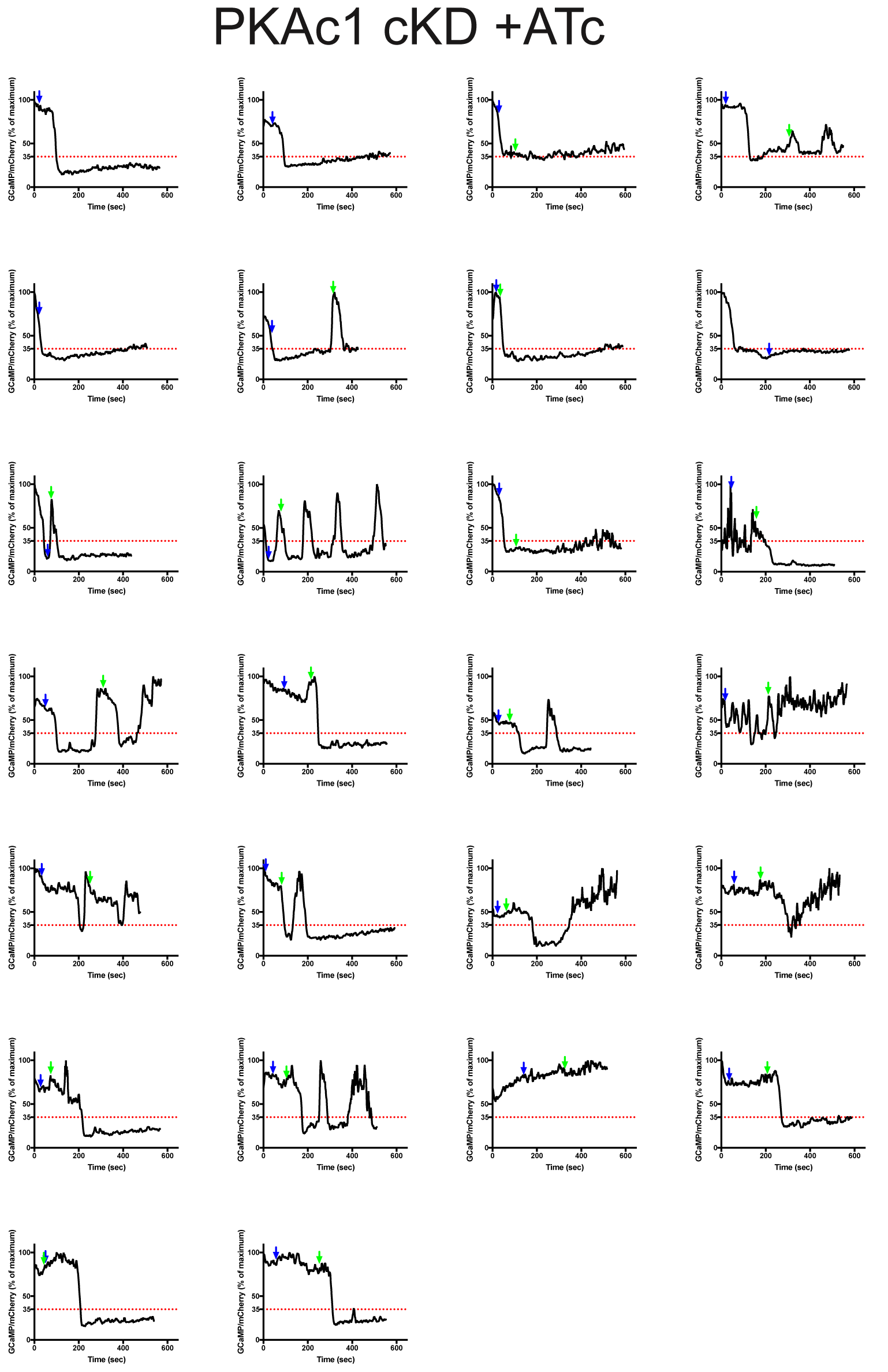

Supplement: S8 Fig — Individual invading tachyzoites were tracked using ImageJ and an intensity ratio between GCaMP6 and mCherry was derived, followed by normalising against the maximum value. Thirty-five percent of maximum is marked with a dotted line to arbitrarily signify ‘baseline’ level. Blue arrow signifies moment of completed invasion. ATc, anhydrotetracycline; cKD, conditional knockdown; GCaMP6, GFP-Calmodulin-M13 peptide-6; PKAc1, protein kinase A catalytic subunit 1. (TIF) [file pbio.2005642.s010.tif]

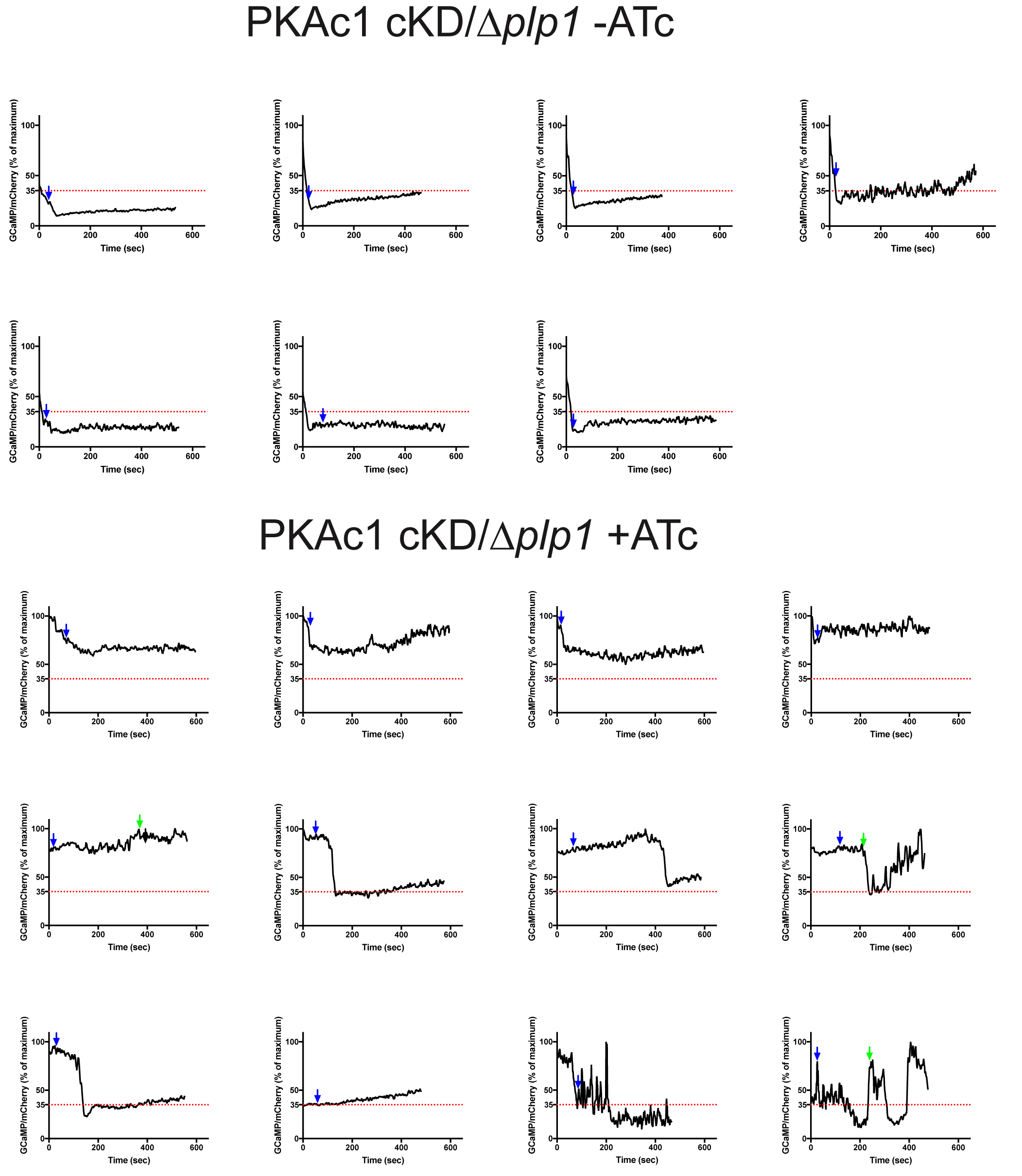

Supplement: S9 Fig — Individual invading tachyzoites were tracked using ImageJ and an intensity ratio between GCaMP6 and mCherry was derived, followed by normalising against the maximum value. Thirty-five percent of maximum is marked with a dotted line to arbitrarily signify ‘baseline’ level. Blue arrow signifies moment of completed invasion and green arrows signifies moment of host cell egress. ATc, anhydrotetracycline; cKD, conditional knockdown; GCaMP6, GFP-Calmodulin-M13 peptide-6; PKAc1, protein kinase A catalytic subunit 1. (TIF) [file pbio.2005642.s011.tif]

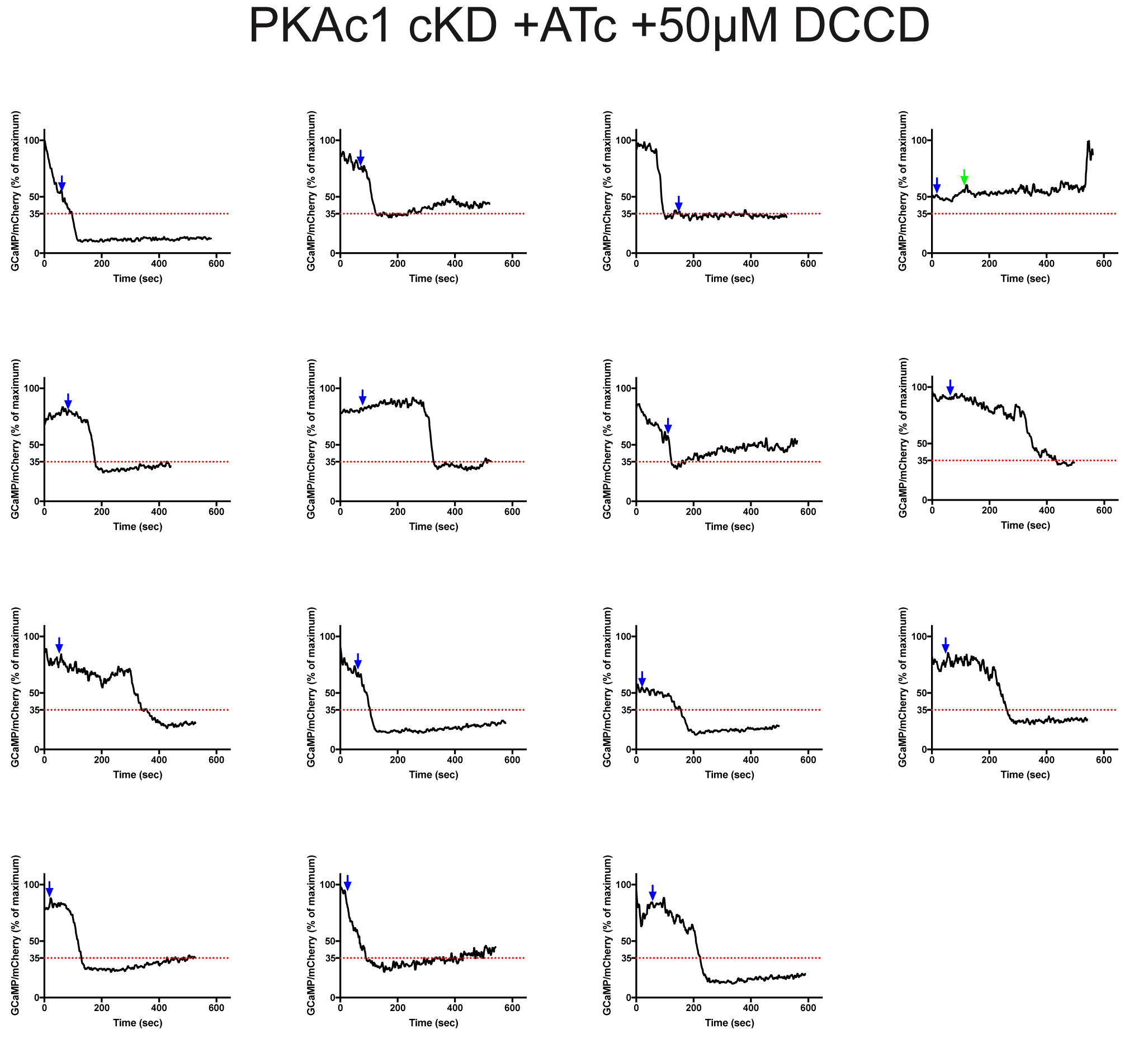

Supplement: S10 Fig — Individual invading tachyzoites were tracked using ImageJ and an intensity ratio between GCaMP6 and mCherry was derived, followed by normalising against the maximum value. Thirty-five percent of maximum is marked with a dotted line to arbitrarily signify ‘baseline’ level. Blue arrow signifies the moment of completed invasion and green arrows signify the moment of host cell egress. cKD, conditional knockdown; DCCD, N,N′-Dicyclohexylcarbodiimide; GCaMP6, GFP-Calmodulin-M13 peptide-6; PKAc1, protein kinase A catalytic subunit 1. (TIF) [file pbio.2005642.s012.tif]

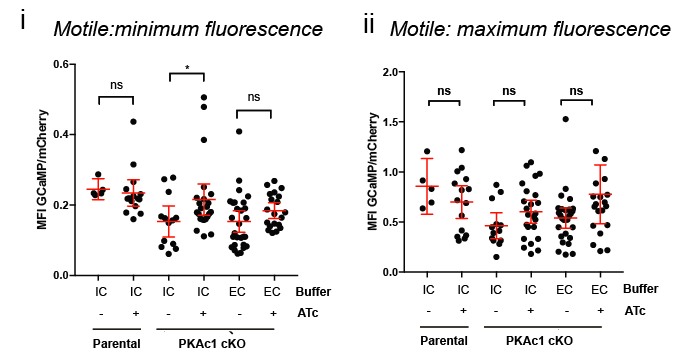

Supplement: S11 Fig — Parental and PKAc1 cKD parasites ±ATc tachyzoites expressing GCaMP6/mCherry were resuspended in either EC or IC buffer and allowed to glide on a glass coverslip. Motile parasites were tracked and maximum and minimum values extracted and compared across conditions. Data represent mean ± SD and P values were calculated by unpaired pairwise t tests. Individual numerical values underlying (i) and (ii) may be found in S1 Data. ATc, anhydrotetracycline; cKD, conditional knockdown; EC, extracellular; GCaMP6, GFP-Calmodulin-M13 peptide-6; IC, intracellular; PKAc1, protein kinase A catalytic subunit 1. (PNG) [file pbio.2005642.s013.png]
