# Supplementary material for: Protein kinase A negatively regulates Ca2+ signalling in Toxoplasma gondii
Source: PLoS Biol. 2018 Sep 12;16(9):e2005642. doi: 10.1371/journal.pbio.2005642 (PMC6152992; doi:10.1371/journal.pbio.2005642)
Supplement: S1 Text — (DOCX) [file pbio.2005642.s027.docx]

**Protein Kinase A Negatively Regulates Ca^2+^ signaling Post- Invasion in *Toxoplasma gondii***

Alessandro D. Uboldi^1,2^, Mary-Louise Wilde^1^, Emi A. McRae^1,2^, Rebecca J. Stewart^1,2^, Laura F. Dagley^1,2^, Luning Yang^1,2,3^, Nicholas J Katris^4,5^, Sanduni V. Hapuarachchi^6^, Michael J Coffey^1,2^, Adele M. Lehane^6^, Cyrille Y Botte^5^, Ross F. Waller^4^, Andrew I. Webb^1,2^, Malcolm J. McConville^7^, Christopher J. Tonkin^1,2*^

1. The Walter and Eliza Hall Institute of Medical Research, Parkville, VIC, Australia

2. Department of Medical Biology, The University of Melbourne, VIC, 3010, Australia

3. School of Medicine, Tsinghua University, Beijing, China.

4. Department of Biochemistry, University of Cambridge, Cambridge, UK.

5. Institute of Advanced Biosciences, CNRS UMR5309, INSERM U1209, Université Grenoble Alpes, Grenoble France

6. Research School of Biology, The Australian National University, A.C.T, Australia

7. Department of Biochemistry and Molecular Biology, Bio21 Molecular Science and Biotechnology Institute, The University of Melbourne, Parkville, VIC, 3010, Australia

^*^Correspondence:

Chris Tonkin

Division of Infection and Immunity

The Walter and Eliza Hall Institute of Medical Research

1G Royal Pde, Parkville

Ph: +613 934 2926

Fax: +613 9347 0852

Email: [tonkin@wehi.edu.au](mailto:tonkin@wehi.edu.au)

**Supplementary materials and methods**

***Sequences of oligonucleotides used in this study are listed in S2 Table***

***Plasmid construction and generation of transgenic lines***

PKAc1 cKD

To make a conditional knockdown for PKAc1 (ToxoDB Gene ID # TGME49_226030), the native promoter was replaced by the anhydrotetracycline (ATc)-responsive T7S4 promoter. Two DNA fragments, corresponding to the 5’ untranslated region of the PKAc1 gene (5’ flank) and the start of the coding region (3’ flank), were amplified from RH genomic DNA by PCR with Prime Star DNA Polymerase (Takara), using oligonucleotides P1 and P2 to amplify the 5’ flank and P3 and P4 for the 3’ flank. The 5’ and 3’ flanks were digested with *Fse*I/*Nsi*I and XmaI/EcoRV, respectively and sequentially ligated to pPR2-3HA vector (G. van Dooren, unpublished) using the same enzymes. The plasmid was linearized with *Fse*I and *Eco*RV I-HF for transfection into the RH:∆*ku80*:TATi strain (a kind gift from Lilach Sheiner and Boris Striepen). Parasites were selected with 1 μM pyrimethamine and cloned by limiting dilution. Successful integration of the construct into the *pkac1* locus was confirmed by using primers P5-10 as shown in S1 Fig.

TyPKAr1

PKAr1 was tagged at the N-terminus after the first 15 amino acids, so as to not disrupt dual acylation and membrane binding. This was achieved by amplifying a homology-directed repair (HDR) template in two steps allowing for the introduction of the 15 amino acid dual acylation sequence, the Ty tag and 5’ and 3’ regions of homology to the PKAr1 gene. This took place by using primers P11 and P12, followed by primers P11 and P13 using pT8TATi-HX-T7S1 plasmid as a template (kindly supplied by D. Sodati-Favre). Integration at the start of the coding sequence proceeded using CRISPR/Cas9 by introducing the guide sequence (ggctctccgtgtgtctgtgg) into pSAG1-Cas9-U6-sgUPRT (kindly supplied by D. Sibley) using primers P14 and P15. HDR template derived by PCR was then purified and co-transfected with guide plasmid into ∆*ku80*:∆*hxgprt* parental line.

PKAr1 N-terminal acylation mutants

PKAr amino acids 1-15 were fused to GFP using long overlapping oligonucleotides that were designed to have sticky ends capable of ligating into *Bgl*II/*Avr*II sites of pHTG [1]. PKAr(1-15) was generated by annealing primers P16 and P17, PKAr1(1-15)_G2A_ using primers P18 and P19, PKAr1(1-15)_C5AC7A_ using primers 20 and 21 and PKAr1(1-15)_G2AC5AC7A_ using primers P22 and P23.

∆*plp1*

To make a construct for deletion of the *PLP1*gene (ToxoDB gene ID # TGME49_204130), DNA regions flanking the gene were amplified from RH genomic DNA. The 5’ flank was amplified using oligonucleotides P24 and P25 and the 3’ flank using primers P26 and P27.

The 3’flank was digested with *Avr*II and ligated to the vector pgCH (a kind gift from Giel van Dooren) via *Spe*I and *Sma*I sites. The resulting plasmid was then digested with *Apa*I and *Xho*I to accept similarly cut PLP1 5’ flank. The PLP1 knockout plasmid was digested with *Dra*III and *Psh*A1 to cut the construct near the boundaries of both flanks and transfected into ∆*ku80*:TATi:pkac1cKD previously generated. Following drug selection with 20 µM chloramphenicol, tachyzoites were cloned by limiting dilution to select clonal Δ*plp1* parasites. Successful integration of the plasmid into the *PLP1* locus was confirmed by using primers P28-33 as outlined in S5 Fig.

**Immunoflourescent assay (IFA)**

Tachyzoites were added to coverslips in 6-well plates containing HFF monolayers and incubated overnight at 37 °C, 10% CO_2_, before removing the medium and fixing with 4% formaldehyde (Sigma) for 20 min at room temperature. Wells were washed once with 2 mL PBS, permeabilized with PBS/0.1% Triton X-100 for 10 min at room temperature, washed once with 2 mL PBS and blocked for at least one hour with PBS/3% BSA, at room temperature. The blocking solution was removed and primary antibodies were added to the wells in PBS/3% BSA and incubated overnight at 4 °C with shaking. The primary antibody was washed off using PBS, before incubating with AlexaFluor-conjugated secondary antibodies (Life Technologies) in the dark, for at least 1 hour at room temperature. The wells were washed four times as before (with the penultimate wash containing 0.2 µg/ml DAPI) and mounted onto slides using Vectashield (Vector Laboratories).

**Host cell invasion assays**

PKAc1KD, PKAc1 KD:∆*plp1* and WT (RH:∆ku80:TATi) parasites from T25 flasks that were approximately 70-90% egressed were scraped and passed through 27-gauge needles, split into two and added to fresh T25 flasks containing confluent HFF monolayers for approximately 4 hours, before washing off uninvaded parasites and replacing the medium with fresh medium ± 1 µg/ml ATc. Parasites were incubated ± ATc for 36 hours, before processing as follows: Host cells/parasites were scraped and passed through 27-gauge needles, before centrifuging at 400 rpm for 5 min (in a Beckman GS-6KR centrifuge) to pellet large cell debris. The resulting supernatants were centrifuged at 2000 rpm for 5 min to pellet parasites. The parasites were washed with IC buffer (25 mM Hepes, pH 7.4; 5 mM NaCl, 142 mM KCl; 2 mM EGTA; 1 mM MgCl_2_; 5.6 mM glucose) and centrifuged as above, before resuspending in IC buffer at 1.3 x 10^6^ parasites/100 µl. Parasites were added to wells of 8-well chamber plates (100 µl/well) (Ibidi) housing confluent monolayers of HFFs that had been washed twice with IC buffer. The parasites were brought into contact with the HFFs by centrifugation at 1300 rpm for 3 min, after which the IC buffer was carefully aspirated and replaced with 200 µl of normal D1 (invasion) medium. The plates were incubated at 37 °C for 10 min to allow invasion to occur, before fixing for 10 min by adding 100 µl of 7.5% formaldehyde/0.06% glutaraldehyde in PBS. The fixative solution was removed and the wells filled with 200 µl of 0.2M Tris, pH 8.0 for 10 min to quench remaining formaldehyde/glutaraldehyde. The wells were washed 3 times with 200 µl PBS and blocked with 3% BSA/PBS for 1 hour at room temperature, before incubating with 1:3000 dilution of mouse anti-SAG1 (DG52) antibody for 1 hour at room temperature to label extracellular parasites. The wells were washed 6 x for 5 min with PBS, before adding 200 µl/well of 0.1% Triton X-100/PBS to the wells for 10 min to permeabilize the cells/parasites. The wells were washed twice with 200 µl PBS, blocked with 3% BSA/PBS for 1hour at room temperature and incubated with 1:2000 dilution of rabbit anti-GAP45 antibody, overnight at 4 °C. Following 4 washes with 200 µl PBS, the wells were incubated with both anti-mouse-Alexa Fluor 488 and anti-rabbit Alexa Fluor 594 secondary antibodies in PBS/3% BSA for 6 hours. The wells were washed 4 times for 5 min with PBS (with the third wash containing DAPI) before imaging the parasites on a Zeiss Live Cell AxioObserver microscope using the 63 X objective. For each biological replicate, 16 panels were imaged, each with an area of 211.3 x 198.1 microns and a z-stack of 4 (8 microns). Counting was done manually using the Cell Counter plugin for Image J. Parasites that were red only (due to GAP45 staining) were counted as intracellular (invaded), while those that were both green (SAG1 staining) and red (GAP45 staining) were counted as extracellular (uninvaded). Parasites that were in the process of invading were considered to be intracellular. The count included parasites on edges. In instances where it was difficult to determine the number of parasites such as when multiple parasites were clumped together, DAPI staining of parasite nuclei was used in addition to fluorescence staining of the parasite periphery to discern the number of parasites.

Invasion assays with DCCD were carried out with PKAc1KD and WT (RH:∆*ku80*:TATi) parasites that also expressed GFP from the *UPRT* locus. In this case, GAP45 staining was not done and SAG1 staining was detected with anti-rabbit Alexa Fluor 594 secondary antibodies. Parasites that were green only (due to GFP expression) were counted as intracellular (invaded), while those that were both green (GFP) and red (SAG1 staining) were counted as extracellular. Parasites that were in the process of invading were considered to be intracellular. Parasites that were red only were not counted as they have lost their GFP fluorescence and therefore are likely dead and unable to invade.

**Host cell Attachment assays**

PKAc1KD and WT (RH:∆ku80:TATi) parasites that also expressed GFP from the *UPRT* locus, were incubated ± ATC and prepared as described above. The parasites were resuspended in D1-Hepes medium at a concentration of 1 x 10^7^ parasites/ml and 200 µl volumes of parasites were added to wells of 8-well chambers (Ibidi) that had been prepared as follows: HFF monolayers were fixed with 2.5% formaldehyde/0.01% glutaraldehyde (final concentrations) for 10 min, treated with 0.2M Tris, pH 8.0 for 7 min and washed three times with PBS. The parasites were brought into contact with the fixed HFF monolayers by centrifugation at 1300 rpm for 3 min and incubated at 37 °C for 30 min to allow attachment to occur, before fixing again with 2.5% formaldehyde/0.01% glutaraldehyde for 20 min. The fixative solution was removed and the wells were filled with 0.2M Tris, pH 8.0 for 7 min. The wells were then washed 3 x with PBS and blocked with 3% BSA/PBS for at least 1 hour at room temperature before proceeding with IFAs as described for the invasion assays above. Parasites that were both green (GFP) and red (SAG1) were considered extracellular and attached, as were parasites that were red (SAG1) only (i.e., even though they have lost their GFP fluorescence and are therefore likely dead, they can still attach to the host cells). The count included parasites on edges. In instances where it was difficult to determine the number of parasites such as when multiple parasites were clumped together, DAPI staining of parasite nuclei was used in addition to fluorescence staining of the parasite periphery.

**Proteomics**

In-gel trypsin digestion and mass spectrometry analysis of HA-tagged PKAc1

Bands of interest were excised and subjected to reduction, alkylation, and trypsin digestion before MS analysis, essentially as previously described h[2]. Briefly, acidified peptide mixtures were analyzed by nanoflow reversed-phase liquid chromatography tandem mass spectrometry (LC-MS/MS) on a nanoAcquity system (Waters), coupled to a Q-Exactive mass spectrometer equipped with a nanoelectrospray ion source for automated MS/MS (Thermo Fisher Scientific). Peptide mixtures (5/20 µl) were loaded on a 20 mm trap column with 180-µm inner diameter (nanoAcquity UPLC 2G-V/MTrap 5 μm Symmetry C18) in buffer A (0.1% formic acid, 99.9% Milli-Q water), and separated by reverse-phase chromatography using a 150-mm column with 75 µm inner diameter (nanoAcquity UPLC 1.7 µm BEH130 C18) on a 60 min linear gradient set at a constant flow rate of 400 nL/min from 5–35% buffer B (0.1% formic acid, 99.9% acetonitrile). The Q-Exactive was operated in a data-dependent mode, switching automatically between one full-scan and subsequent MS/MS scans of the ten most abundant peaks. The instrument was controlled using Exactive series version 2.8 build 2806 and Xcalibur 4.0. Full-scans (*m*/*z* 350–1,850) were acquired with a resolution of 70,000 at 200 *m*/*z*. The 10 most intense ions were sequentially isolated with a target value of 10000 ions and an isolation width of 2 *m*/*z* and fragmented using HCD with normalized collision energy of 19.5, 26, 32.5. Maximum ion accumulation times were set to 50 ms for full MS scan and 200 ms for MS/MS. Underfill ratio was set to 5% and dynamic exclusion was enabled and set to 90 s.

Detection for changes in microneme secretion of PKAc1 mutants using SILAC labelling

To look for quantitative differences in secretion of micronemes, parental (∆*ku80*:TATi and ∆*ku80*:TATi:*pkac1* cKD, tachyzoites were inoculated onto fresh host cells and allowed to grow for one lytic cycle. Freshly-lysed tachyzoites were then grown in DMEM medium for SILAC (Thermo Fisher), supplemented to 1% v/v with dialysed foetal calf serum (FCS) (Invitrogen) and 131 mg/L L-Leucine (Sigma), then for light labelling: 183 mg/L L-Lysine (Sigma) and 75 mg/L L-Arginine (Sigma); and for heavy labelling: 183 mg/L Lys8 and 75 mg/L Arg10 (Sigma). Parasites were passaged for approximately a week to maximize labelling, then equal numbers were counted and seeded onto confluent host cells with the addition of 1µg/ml of ATc. At 24 hours post infection, parasites were harvested for a microneme secretion assay in a final volume of 2 mL. Equal supernatant volumes of reciprocally paired samples, e.g. ∆*ku80*:TATi +ATc (heavy) and PKAc1 +ATc (light) and ∆*ku80*:TATi +ATc (light) and PKAc1 +ATc (heavy), were mixed, then concentrated using a 5 kDa molecular weight cut off Vivaspin 20 column (Sartorius) until they reached a final volume of 400 µL.

Trypsin digestion

SILAC-labelled supernatants were denatured with urea powder to 6M final concentration. Eluates from HA-PKAc1 IPs (*n* = 3 per group) and SILAC-labelled supernatants were prepared for mass spectrometry analysis using the FASP protein digestion method as previously described [3] with the following modifications. Protein material was reduced with Tris-(2-carboxyethyl)phosphine (TCEP, 10 mM final concentration). Eluates were digested with sequence-grade modified Trypsin Gold (Promega, V5280) (2 µg) in 50 mM ammonium bicarbonate (NH_4_HCO_3_) and incubated overnight at 37 °C. Peptides were then eluted with 50 mM NH_4_HCO_3_ in two 40 µL sequential washes and acidified in 1% formic acid (final concentration). The peptides were then lyophilised to dryness using a CentriVap (Labconco) prior to reconstituting in 75 µL Buffer A (0.1% FA/2% ACN) ready for MS analysis.

Mass spectrometry analysis

Peptides derived from IPs (2 µl) and SILAC-labelled supernatants (3 µl) were separated by reverse-phase chromatography on a 1.9 µm C_18_ fused silica column (I.D. 75 µm, O.D. 360 µm x 25 cm length) packed into an emitter tip (Ion Opticks, Australia), using a nano-flow HPLC (M-class, Waters). SILAC-labelled supernatants were run in technical triplicate. The HPLC was coupled to an Impact II UHR-QqTOF mass spectrometer (Bruker, Bremen, Germany) using a CaptiveSpray source and nanoBooster at 0.20 Bar using acetonitrile. Peptides were loaded directly onto the column at a constant flow rate of 400 nL/min with buffer A (99.9% Milli-Q water, 0.1% formic acid) and eluted with a 90 min linear gradient from 2 to 34% buffer B (99.9% acetonitrile, 0.1% formic acid). Mass spectra were acquired in a data-dependent manner including an automatic switch between MS and MS/MS scans using a 1.5 second duty cycle and 4 Hz MS1 spectra rate followed by MS/MS scans at 8-20 Hz dependent on precursor intensity for the remainder of the cycle. MS spectra were acquired between a mass range of 200–2000 m/z. Peptide fragmentation was performed using collision-induced dissociation (CID).

Data analysis

Raw files consisting of high-resolution MS/MS spectra were processed with MaxQuant (version 1.5.3.30) for feature detection and protein identification using the Andromeda search engine [3]. Extracted peak lists were searched against the *Toxoplasma gondii* ME49 (ToxoDB-26.0) database as well as a separate reverse decoy database to empirically assess the false discovery rate (FDR) using strict trypsin specificity allowing up to 2 missed cleavages. The minimum required peptide length was set to 7 amino acids. In the main search, precursor mass tolerance was 0.006 Da and fragment mass tolerance was 40 ppm. The search included variable modifications of oxidation (methionine), amino-terminal acetylation, the addition of pyroglutamate (at N-termini of glutamate and glutamine) and a fixed modification of carbamidomethyl (cysteine). For the SILAC searches, fixed Arg4 and Lys4 modifications were enabled. The “match between runs” option in MaxQuant was used to transfer identifications made between runs within a group of samples on the basis of matching precursors with high mass accuracy [4]. PSM and protein identifications were filtered using a target-decoy approach at an FDR of 1%.

Label-free quantitative proteomics pipeline (PKA IPs)

Statistically-relevant protein expression changes between the PKAc1 and control IPs were identified using a custom in-house designed pipeline as previously described [5], where quantitation was performed at the peptide level. Probability values were corrected for multiple testing using Benjamini–Hochberg method. Cut-off lines with the function y= -log_10_(0.05)+c/(x-x_0_) [6] were introduced to identify significantly enriched proteins. c was set to 0.2 while x_0_ was set to 1, representing proteins with a twofold (log2 protein ratios of 1 or more) or fourfold (log2 protein ratio of 2) change in protein expression, respectively.

SILAC quantitative proteomics pipeline (supernatants)

Analysis of the SILAC MaxQuant output was performed using a custom pipeline developed in Pipeline Pilot (Biovia), which takes as input the MaxQuant output files allPeptides.txt, peptides.txt and evidence.txt. A feature was defined as the combination of peptide sequence, charge and modification. Features not found in at least half the number of replicates in each group were removed. Proteins identified from hits to the reverse database and proteins with only one unique peptide were also removed. The probability of differential expression between groups was calculated using the Wilcoxon Rank Sum test excluding any non-unique sequences and any features with modifications other than oxidation and carbamidomethylation.

**References:**

1. McCoy JM, Whitehead L, Van Dooren GG, Tonkin CJ. TgCDPK3 regulates calcium-dependent egress of *Toxoplasma gondii* from host cells. PLoS Pathog. 2012;8: e1003066. doi:10.1371/journal.ppat.1003066

2. Hildebrand JM, Tanzer MC, Lucet IS, Young SN, Spall SK, Sharma P, et al. Activation of the pseudokinase MLKL unleashes the four-helix bundle domain to induce membrane localization and necroptotic cell death. Proceedings of the National Academy of Sciences. 2014;111: 15072–15077. doi:10.1073/pnas.1408987111

3. Wisniewski JR, Zougman A, Nagaraj N, Mann M. Universal sample preparation method for proteome analysis. Nat Meth. 2009;6: 359–362. doi:10.1038/nmeth.1322

4. Cox J, Mann M. MaxQuant enables high peptide identification rates, individualized p.p.b.-range mass accuracies and proteome-wide protein quantification. Nat Biotechnol. 2008 ed. 2008;26: 1367–1372. doi:10.1038/nbt.1511

5. CIS is a potent checkpoint in NK cell-mediated tumor immunity. Nature Publishing Group; 2016. doi:10.1038/ni.3470

6. Keilhauer EC, Hein MY, Mann M. Accurate protein complex retrieval by affinity enrichment mass spectrometry (AE-MS) rather than affinity purification mass spectrometry (AP-MS). Molecular & Cellular Proteomics. American Society for Biochemistry and Molecular Biology; 2015;14: 120–135. doi:10.1074/mcp.M114.041012

7. Kessler H, Herm-Götz A, Hegge S, Rauch M, Soldati-Favre D, Frischknecht F, et al. Microneme protein 8--a new essential invasion factor in *Toxoplasma gondii*. Journal of Cell Science. 2008;121: 947–956. doi:10.1242/jcs.022350

8. Muzumdar MD, Tasic B, Miyamichi K, Li L, Luo L. A global double-fluorescent Cre reporter mouse. genesis. 2007;45: 593–605. doi:10.1002/dvg.20335
